# *Giardia* spp.-induced microbiota dysbiosis disrupts intestinal mucin glycosylation

**DOI:** 10.1080/19490976.2024.2412676

**Published:** 2024-10-16

**Authors:** Elena Fekete, Thibault Allain, Olivia Sosnowski, Stephanie Anderson, Ian A. Lewis, Andre G. Buret

**Affiliations:** a Department of Biological Sciences, University of Calgary, Calgary, Canada; b Host-Parasite Interaction Network, University of Calgary, Calgary, Canada; c Inflammation Research Network, University of Calgary, Calgary, Canada

**Keywords:** *Giardia*, mucus, glycans, microbiota, dysbiosis

## Abstract

Infection with the protozoan parasite *Giardia duodenalis* (syn. *intestinalis*, *lamblia*) has been associated with intestinal mucus disruptions and microbiota dysbiosis. The mechanisms remain incompletely understood. Mucus consists primarily of densely glycosylated mucin glycoproteins. Mucin *O*-glycans influence mucus barrier properties and mucin–microbe interactions and are frequently altered during disease. In this study, we observed time-dependent and regiospecific alterations to intestinal mucin glycosylation patterns and the expression of mucin-associated glycosyltransferase genes during *Giardia* infection. Glycosylation alterations were observed in *Giardia*-infected mice in the upper small intestine, the site of parasite colonization, and in the distal colon, where active trophozoites were absent. Alterations occurred as early as day 2 post-infection and persisted in mice after parasite clearance. We also observed small intestinal goblet cell hyperplasia and thinning of the distal colon mucus barrier during early infection, and microbiota alterations and altered production of cecal SCFAs. *Giardia*-induced alterations to mucin glycosylation were at least in part dependent on microbiota dysbiosis, as transplantation of a dysbiotic mucosal microbiota collected from *Giardia*-infected mice recapitulated some alterations. This study describes a novel mechanism by which *Giardia* alters intestinal mucin glycosylation, and implicates the small intestinal microbiota in regulation of mucin glycosylation patterns throughout the gastrointestinal tract.

## Introduction

The major component of intestinal mucus is the secreted glycoprotein mucin 2 (MUC2). Mucins like MUC2 are highly glycosylated via the addition of *O*-linked glycans to serine and threonine residues within the protein core. *O*-glycosylation regulates mucin biochemistry, mucus barrier structure and function, and mucin–microbe interactions.^1–3^*O*-glycosylation can protect the mucin from degradation by the host and many microbial proteases, and mucin glycans may be utilized as binding sites by commensal microbes to facilitate colonization of the gut and to prevent translocation through the underlying epithelium.^4,5^ Mucin glycans may also be bound or modified by pathogenic microbes to initiate infection,^4,5^ or degraded as a nutrient source.^2,5,6^ Excessive mucin degradation may result in loss of barrier function and increased intestinal permeability, while competition for mucin-derived nutrients during infection may contribute to dysbiosis.^4,6,7^ As a result, alterations to glycosylation patterns are frequently observed in association with infection, dysbiosis, and inflammation, although precise cause-to-effect mechanisms remain poorly understood.^1,2,4^

Up to 80% of the weight-by-volume of MUC2 is attributed to *O*-linked glycans.^3^ MUC2 polymerizes extensively via disulfide bonding between cysteine-rich *N*- and C-termini, forming a complex network that, when hydrated, forms a viscous gel overlying the intestinal epithelium. In the small intestine, a non-sterile single layer of loosely adherent mucus composed of both Muc2 and Muc5ac harbors high concentrations of antimicrobial molecules to maintain barrier function. In the colon, a thick double layer of mucus composed predominantly of Muc2 consists of a sterile and firmly adherent inner layer and a less dense and non-adherent outer layer that is colonized by the commensal microbiota.^8^

Mucin *O*-glycosylation is mediated by glycosyltransferase enzymes whose expression is regulated by both host and environmental factors including genetics, diet, the microbiota, and the immune system. MUC2 *O*-glycosylation is initiated with the addition of N-acetylgalactosamine (GalNAc) to the hydroxyl groups of serine and threonine residues by 1 of 20 UDP-GalNAc:polypeptide *N*-acetylgalactosaminyl transferases (GalNAc-Ts) to form the Tn antigen. Addition of Gal to the Tn antigen by C1GalT1 forms the core 1 structure, while addition of N-acetylglucosamine (GlcNAc) by C3GnT forms core 3 structures. Core 1 and core 3 structures can be modified to form core 2 and core 4 structures, respectively, via addition of GlcNAc by C2GnT1, C2GnT2, or C2GnT3. Core structures are then further extended via addition of Gal, GalNAc, and GlcNAc to form complex polysaccharide chains of variable lengths. Mucin *O*-glycans are typically capped with sialic acid, fucose, and/or sulfate groups, whose addition is catalyzed by sialyltranferases, fucosyltransferases, and sulphotransferases, respectively.^3,9^

The waterborne protozoan parasite *Giardia duodenalis* is a leading cause of diarrheal disease worldwide. Upon ingestion, cysts begin the process of excystation, initiated in the stomach with exposure to acidic pH and host proteases. This process is completed in the upper small intestine, and results in the release of two vegetative trophozoites per cyst. *Giardia* infections may cause diarrhea, abdominal pain, nausea, and intestinal malabsorption, although asymptomatic infections are also common. *Giardia* has also been associated with post-infectious complications including post-infectious irritable bowel syndrome, chronic fatigue, and stunted growth and failure to thrive in children.^10^

*Giardia* infections, as well as other intestinal disorders such as inflammatory bowel disease (IBD) and irritable bowel syndrome (IBS), are associated with both microbiota dysbiosis and increased intestinal permeability. Previous research has established that, during acute *Giardia* infection *in vitro* and *in vivo*, goblet cell activity and the expression and secretion of intestinal mucins is altered.^11,12^ These alterations are mediated by *Giardia* cysteine protease activity. The aim of this study was to investigate whether and how *Giardia* infection may alter mucin glycosylation patterns. As alterations to glycosylation patterns and dysbiosis are closely linked, we investigated the role of the small intestinal microbiota in modulating goblet cell activity in both the small and large intestines using a novel technique of small intestinal microbiota transplant. The findings reveal that *Giardia* infection disrupts mucin glycosylation and the expression of mucin-associated glycosyltransferases in the small and large intestines, at least in part in a manner dependent on a dysbiotic microbiota.

## Methods

### In vivo *experimental design*

All experimental protocols were approved by the University of Calgary Animal Care Committee (Protocol #AC21-0055) in accordance with Canadian Council on Animal Care guidelines. *Giardia muris* was maintained via routine passage through C57BL/6 mice. *G. duodenalis* isolate GS/M was purchased from ATCC. *G. duodenalis* isolate NF was isolated from a giardiasis outbreak in Newfoundland, Canada.^13^

To prepare *G. muris* trophozoites, infected mice were anaesthetized and sacrificed by cervical dislocation, and a 3 cm length of the proximal small intestine was collected. Proximal small intestines were incubated on ice with frequent vortexing to detach trophozoites, and trophozoites were counted using a hemocytometer and diluted to appropriate concentrations in sterile PBS. *G. duodenalis* was cultured axenically in 15 mL polystyrene conical tubes with Keister’s modified TYI-S-33 medium^14^ supplemented with penicillin–streptomycin (Sigma-Aldrich). At peak culture density, tubes were placed on ice for 20 min and vortexed to detach trophozoites. Trophozoites were isolated via centrifugation for 10 min at 1500 rpm, washed with sterile PBS, then re-suspended in PBS or cell culture media, counted using a hemocytometer, and diluted to appropriate concentrations for each assay.

Three-four-week-old male and female C57BL/6 mice obtained from the LESARC colony (University of Calgary) were infected with *Giardia muris* trophozoites (5 × 10^4^ trophozoites in 100 µL PBS) or *Giardia duodenalis* isolate GS/M trophozoites (5 × 10^7^ trophozoites in 100 µL PBS) via oral gavage, or gavaged with PBS as a vehicle control. At day 2, day 7, and day 30 post-infection (PI), mice were weighed, intestinal transit was measured as below, and feces were collected. To measure intestinal transit, mice were placed in individual cages without access to food or water. Over the course of one hour, the number of fecal pellets produced per mouse was counted. A total of 1–2 fecal pellets were collected, weighed, and dehydrated overnight. The weight of the hydrated versus dehydrated pellet was compared to quantify the fecal water content.

**Figure 1.**
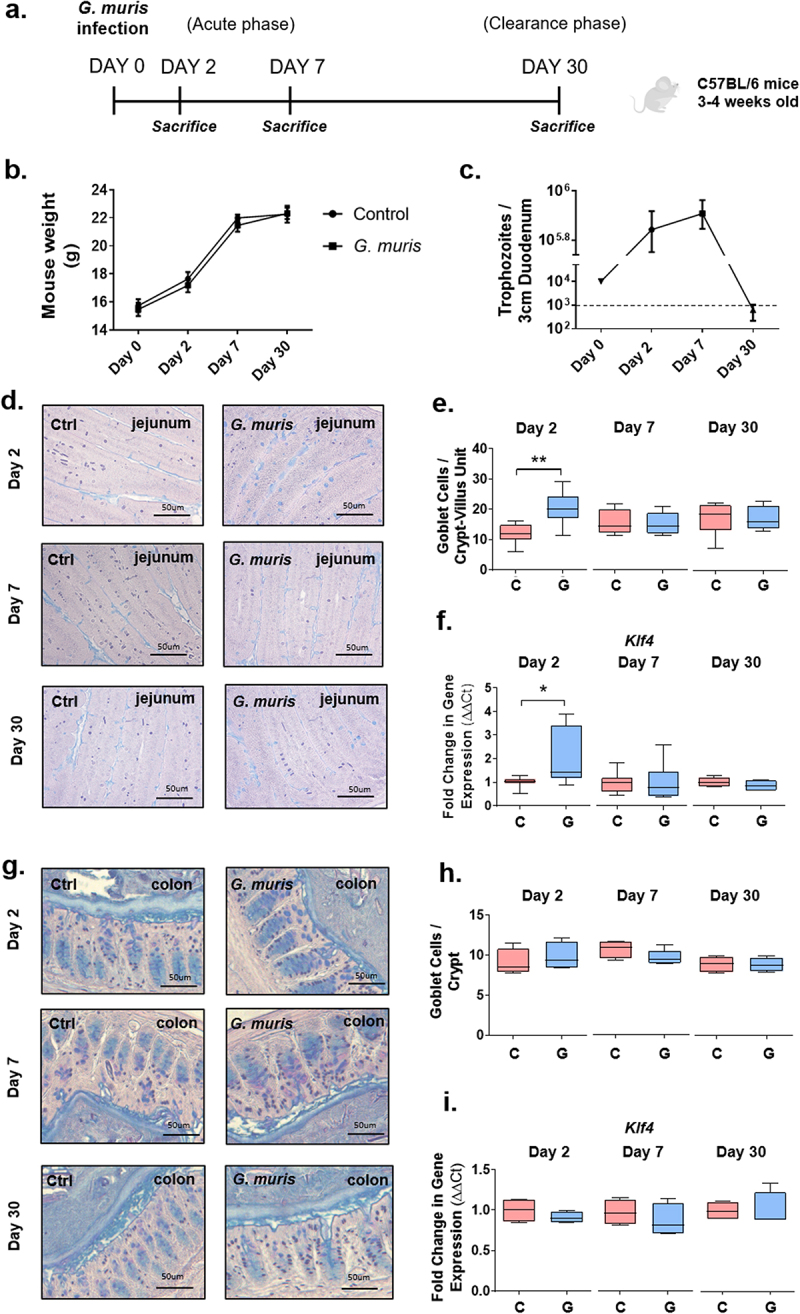
*Giardia* infection induces goblet cell hyperplasia in the small intestine. (a) experimental design. 3-4-week-old wild-type C57BL/6 mice were infected with 5 × 10^4^ *Giardia muris* trophozoites per mouse for 2, 7, or 30 days. At each timepoint, mice were euthanized and tissues and feces were collected. (b) Mouse weight (grams) in control and infected mice at day 2, day 7, and day 30 post-infection. *n* = 8. (c) Trophozoite burden in a 3 cm segment of proximal duodenum. The lower limit of detection is indicated by the dashed line. *n* = 8. (d) jejunum sections from C57BL/6 mice infected with *G. muris* trophozoites for 2, 7, and 30 days were stained with periodic acid/alcian blue staining to detect mucin-filled goblet cells. (e) Goblet cells in the jejunum were counted per crypt-villus unit. *n* = 8. (f) Expression of the goblet cell specific differentiation factor Klf4 was measured using qPCR and a fold change in mRNA expression was calculated compared to an uninfected control average at each timepoint. *n* = 4-8. (g) Colon sections from C57BL/6 mice infected with *G.* *muris* trophozoites for 2, 7, and 30 days were stained with periodic acid/alcian blue staining. (h) goblet cells in the distal colon were counted per crypt. *n* = 4-5. (i) Expression of Klf4 was measured using qPCR and fold change in mRNA expression was calculated compared to an uninfected control average at each timepoint. *n* = 4. Data are shown as box plots (median and interquartile range) with min/max whiskers. *indicates *p* < 0.05, **indicates *p* < 0.01 (student’s T-test).

Mice were anaesthetized and sacrificed via cervical dislocation, and tissues were collected for analysis (Figure 1a). The proximal duodenum was collected in order to assess trophozoite burden and confirm infection. The duodenum was opened longitudinally, incubated in PBS on ice for 20 min, and vortexed to detach adherent trophozoites. Trophozoites were counted using a hemocytometer. Segments (1 cm) of jejunum and distal colon were snap frozen for RNA extraction or fixed in formalin or Carnoy’s fixative for histology.

### Periodic acid/alcian blue staining and goblet cell counts

Sections (5 µm) of jejunum and colon were warmed at 60°C for 10 min, deparaffinized via 3 changes of xylene (3 min each), and rehydrated via 2 changes each of 100% ethanol and 95% ethanol (10 dips each), and 5 min in running tap water. Periodic Acid Schiff’s and Alcian Blue staining were performed according to Newcomer Supply PAS/AB kit protocols. Goblet cells were counted in the jejunum for 8–10 crypt-villus units per mouse, and in the colon in 8–10 crypts per mouse at 20X magnification, then averaged per mouse, as described previously.^11^

### Quantitative PCR

1 cm segments of jejunum or distal colon were added to 600 µL of RLT buffer (Qiagen) supplemented with 0.1% β-mercaptoethanol in a 2 mL homogenization tube containing 1 small metal bead. Tissues were homogenized using a beat beater (3 × 30 seconds at 4.5 m/s). Samples were centrifuged for 10 min at 8000 rpm, and supernatants were collected and mixed with a 1:1 volume of 70% ethanol. Supernatants were added to RNA purification columns (Enzymax), and RNA was extracted according to Qiagen RNEasy mini kit protocols. RNA was quantified via Nanodrop and reverse transcribed according to Fisher Scientific High-Capacity cDNA Reverse Transcription Kit protocols. Quantitative PCR (qPCR) was performed using Qiagen Quantitect SYBR Green kits according to manufacturer protocols. Primer sequences are listed in Supplementary Table 1.

### MUC2 antibody staining

Tissue sections were deparaffinized as above. Sections were blocked for 1 hour with 1% bovine serum albumin (BSA) (Sigma) in 1X PBS + 0.1% Tween-20 (PBST). Sections were incubated overnight at 4°C with an anti-MUC2 antibody (Abcam EPR23479–47) at a 1:100 dilution in 1% BSA. Slides were incubated with alexafluor 488 donkey anti-rabbit secondary antibody (Invitrogen A21206) at a 1:500 dilution in PBST for 1 hour, washed with PBST followed by PBS, then mounted with fluoroshield containing DAPI (Sigma-Aldrich). Tissue sections were imaged, and fluorescence was quantified using ImageJ software. Total fluorescence was quantified at 20X magnification, normalized to tissue area, and averaged for 3–4 images per mouse. Mucus thickness and MUC2 staining were quantified only on samples with intact mucus layers. Average fluorescence was quantified for uninfected controls, and a fold change in fluorescence was calculated by comparing each mouse to the control average.

### High-iron diamine/alcian blue staining and goblet cell counts

Sections (5 µm) of jejunum or colon were deparaffinized as above. Slides were incubated for 20 hours in a fresh HID reagent. HID reagent was prepared by mixing 50 mL solution A (120 mg N,N-dimethyl-meta-phenylenediamine (Fisher Scientific), 20 mg N,N-dimethyl-para-phenylenediamine (Sigma), 50 mL distilled water) with 1.4 mL solution B (40 g ferric chloride (Sigma), 5% hydrochloric acid, 90 mL distilled water). Slides were then washed and incubated in 1% alcian blue in 3% acetic acid (Newcomer Supply) for 30 min, followed by 1% aqueous periodic acid (Newcomer Supply) for 10 min. Slides were then incubated in Schiff’s reagent (Newcomer Supply) for 10 min and washed 3 × 3 min in 0.5% sodium metabisulfite solution (Sigma-Aldrich). Slides were dehydrated via 2 changes each of 95% ethanol and 100% ethanol and 3 changes of xylene (10 dips each), then mounted with polymount.

Predominantly sulfomucin positive goblet cells (stained purple/black) and predominantly sialomucin positive goblet cells (stained blue) were counted in the jejunum per crypt-villus unit and in the colon per crypt at 20X magnification, then averaged for 8–10 crypts or crypt-villus units per mouse.

### Staining with fluorescein-coupled lectins

Tissue sections were deparaffinized as above, then blocked for 30 min in 1X carbo-free blocking solution (Fisher Scientific) supplemented with 0.05% Tween-20. Sections were stained with 10 µg/mL fluorescein-coupled lectins (all purchased from Vector Laboratories) in 1X PBS for 30 min then mounted with fluoroshield containing DAPI. Wheat germ agglutinin (WGA) was used to detect GlcNAc, dolichos biflorus agglutinin (DBA) was used to detect GalNAc, peanut agglutinin (PNA) was used to detect galactosyl(β-1,3)N-acetylgalactosamine, sambucus nigra (SNA) was used to detect α-2,3 and α-2,6 sialic acid, and ulex europaeus (UEA-1) was used to detect α-fucose. Fluorescence was quantified at 20X magnification using ImageJ, normalized to the total tissue area, and averaged for 3–4 images per mouse. The fold change in fluorescence was calculated as for Muc2 staining.

### Fecal DNA extraction and 16S sequencing

Whole bacterial genomic DNA extraction procedure was adapted from Lamas et al. and Allain et al.^15,16^ Mouse fecal pellets collected at each timepoint were thawed in 250 µl of 4 M guanidine thiocyanate in tris-HCl (0.1 M, pH 7.5) with 40 µl of N-laurosyl sarcosine (10%) (Sigma Aldrich). 500 µL N-laurosyl sarcosine (5%) in phosphate buffer (0.1 M, pH 8) was added, and samples were incubated at 70°C for 2 hours. Silica glass beads (0.1 mm, 500 mg) were added, and samples were homogenized using a Fastprep bead beater (6.5 m/s, 3 × 30 seconds with 5-min breaks on ice between runs). 15 mg of polyvinylpolypyrrolidone (PVPP) (Sigma Aldrich) was added and tubes were centrifuged for 5 min at 20,000 *g*. Supernatants were collected and pellets were washed twice with TENP buffer (Tris-Cl, 0.5 M EDTA, 5 M NaCl, 1% PVPP). Supernatants from each wash step were combined and centrifuged to remove the remaining debris. Ice-cold isopropanol was added, and samples were incubated at room temperature for 10 min. Samples were centrifuged at 20,000 *g* for 10 min, then pellets were re-suspended in 450 µL phosphate buffer (0.1 M, pH 8) and 50 µl potassium acetate (5 M) and incubated at 60°C for 10 min. Samples were incubated overnight at 4°C, then centrifuged at 20,000 *g* for 30 min. Samples were incubated with 2 µl RNAse (10 mg/mL, Thermofisher Scientific) at 37°C for 30 min. 1 mL of ice-cold absolute ethanol and 50 µl of 3 M sodium acetate were added to precipitate DNA, and samples were centrifuged at 20,000 *g* for 10 min. Pellets were washed with 70% ethanol, dried at RT, and resuspended in 1X TE buffer. DNA was further purified using a DNA clean and concentrator kit (Zymo research).

Amplicon sequencing libraries were obtained from the V4 region of the 16S SSU rRNA. Paired-end amplicons (250 bases) were sequenced on Illumina Mi-Seq PE250 (Genome Québec Innovation Centre, Montreal, Canada). Bioinformatics analyses were performed using QIIME and Microbiome analyst online software. The 16S rRNA amplicons were clustered into operational taxonomic units (OTU) with a 97% identity threshold. Data are represented using Total Sum Scaling (proportional abundance of species) to remove sequencing-related technical biases, and data were neither rarefied nor transformed. Samples that failed to pass Quality Control step and/or samples with low abundance in OTUs were removed from the dataset. The β-diversity among microbial communities was assessed using the Bray-Curtis dissimilarity index and visualized through Principal Coordinate Analysis (PCoA) plot. The α-diversity was calculated using either Shannon or Simpson diversity index. Linear Discriminant Analysis Effect Size was used to characterize bacterial taxa biomarkers for each condition at the family level (LDA score).

### Small intestinal microbiota transplants

Male and female C57BL/6 mice (3–4-week-old) were infected with 10^4^ *G. muris* trophozoites as above. At day 7 PI, mice were anaesthetized and sacrificed, and the entire length of the small intestine was collected. The small intestine was opened longitudinally, placed in 10 mL sterile PBS, and vortexed. The solution was filtered through gauze to remove tissue and bulk fecal matter, then filtered through a 100 µm filter, a 40 µm filter, and a 5 µm filter (Corning) to isolate the microbiota. Microbes were pooled from four mice per group. The absence of *Giardia* trophozoites was confirmed visually using a light microscope. Optical density was measured to determine microbial concentrations, and concentrations were normalized between control and infected mouse-derived bacterial preparations. Three–four-week-old male and female C57BL/6 mice were fasted for 1 hour with free access to water. Mice were then gavaged with 150–200 µL of polytheylene glycol (PEG, 425 g/L) (Sigma) 4 times at 20-min intervals to remove the indigenous microbiota (previously validated by Wrzosek et al., 2018).^17^ Mice were again transferred to clean cages to avoid coprophagia of left-over fecal pellets, and 4 hours after the final PEG treatment, were gavaged with 200 µL of small intestinal microbiota isolated from either control or *G. muris* infected mice. Seven days after microbiota transplant, intestinal transit was measured, feces were collected, mice were sacrificed, and tissues were collected and analyzed as described above. The duodenums of transplant recipient mice were collected, and the absence of *Giardia* trophozoites was confirmed visually, as described above for trophozoite counts. Fecal microbiota DNA extraction, Illumina 16S sequencing, and data analysis methods for microbiota characterization were performed as described above.

### Short-chain fatty acid quantification

Cecal Short-Chain Fatty Acids (SCFAs) were measured by liquid chromatography-mass spectrometry (LC-MS). Ceca from control, *G. muris* infected, and microbiota transplant recipient mice were collected and snap-frozen prior to analysis. Ceca were opened, and cecal contents were collected and weighed. Cecal contents were added to ice-cold extraction solvent in a 1:2 weight (mg):volume (µl) ratio and homogenized at 30 hz for 3 min. Samples were centrifuged at 18,000 *g* for 10 min, and supernatants were collected and transferred to deep well plates (Axygen #P-DW-20-C-S). Plates were sealed with aluminum adhesive seal (Nunc sealing tapes, Thermo Scientific). Plates were centrifuged at 4000 rpm for 10 min, and supernatants were collected and transferred to a MS 96 well plate (Greiner V bottom, #651291). 2.4 M aniline solution in acetonitrile was added (5 µl per 100 µl sample), and 1.2 M EDC solution in H_2_O was added (5 uL per 100 uL sample). Samples were mixed and incubated on ice for 2 hours. Samples were diluted in 50% methanol and transferred to a MS 96 well plate for LC-MS analysis at the Calgary Metabolomics Research Facility (CMRF). SCFAs were quantified using an isotope-based strategy for absolute quantification of SCFAs (SQUAD method) adapted from Bihan et al.^18^

## Human cell culture and in vitro G. duodenalis infection

LS174T human goblet cells (ATCC CL-188) were grown to confluency in the Minimum Essential Medium Eagle (MEME) (Sigma-Aldrich) supplemented with penicillin–streptomycin, L-glutamine, sodium pyruvate (all purchased from Sigma-Aldrich) and 10% heat-inactivated fetal bovine serum (Gibco, USA). Cells were infected with *G. duodenalis* isolate NF (Assemblage A) or GS/M (Assemblage B) trophozoites at 10:1 multiplicity of infection (MOI) in MEME and incubated for 3 hours at 37°C. Following incubation, cells were washed 3 times with ice-cold PBS to remove adherent trophozoites. Cells were lysed in RLT buffer containing β-mercaptoethanol and RNA was extracted according to Qiagen RNEasy mini kit protocols, as described above. cDNA was synthesized using Fisher Scientific High-Capacity cDNA Reverse Transcription Kit protocols. Quantitative PCR (qPCR) was performed using Qiagen Quantitect SYBR Green kits according to manufacturer protocols. Primer sequences are listed in Supplementary Table S2.

### Statistical analysis

Statistical analysis was performed using Graphpad Prism software (Graphpad Prism 8, La Jolla, USA). Normality of the data was assessed prior to statistical analysis. A one-way analysis of variance (ANOVA) followed by a Tukey’s test for multiple comparisons was used to compare three or more groups of normally distributed data. A student’s T-test was used to compare two groups of parametric data. A Mann–Whitney test was used to compare two sets of non-parametric data. *p* values of less than 0.05 were considered statistically significant.

## Results

### Giardia *infection does not alter mouse weight gain, intestinal transit, or fecal water content*

In the present study, *G. muris* infection (Figure 1a) did not impair mouse weight gain over 30 days (Figure 1b). Similarly, the number of fecal pellets produced over 1 hour and fecal water content per fecal pellet were not different between groups at any timepoint (Sup. Figure S1). Trophozoite burden increased in comparison to the day 0 inoculum at both day 2 and day 7, indicating successful establishment of infection, and 6 of 8 infected mice had successfully cleared the infection by day 30 PI (Figure 1c).

### Giardia *infection induces intestinal goblet cell hyperplasia and reduces mucus layer thickness*

**Figure 2.**
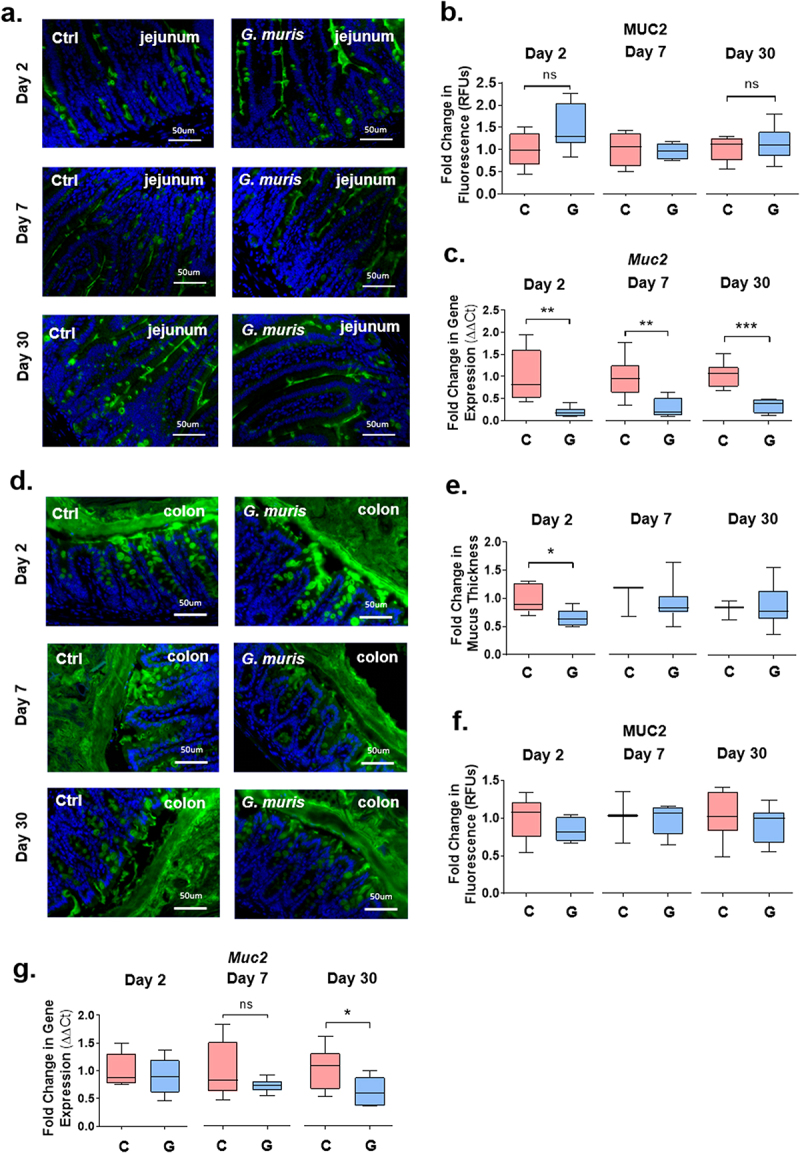
*Giardia* infection causes thinning of the colonic mucus layers and altered mucin gene expression in jejunum and colon. (a) Jejunum sections from C57BL/6 mice infected with *Giardia muris* trophozoites for 2, 7, and 30 days were stained with a MUC2 antibody. (b) Total fluorescence was calculated and normalized to tissue section area to quantify MUC2 protein abundance. A fold change compared to an uninfected control average was calculated for each timepoint. *n* = 4-8. (c) *Muc2* gene expression in the jejunum was measured using qPCR and a fold change in expression was calculated compared to an uninfected control average at each timepoint. *n* = 8. (d) Colon sections from C57BL/6 mice infected with *Giardia muris* trophozoites for 2, 7, and 30 days were stained with a MUC2 specific antibody. (e) Thickness of the inner colonic mucus layer was measured in 10-12 regions of each section when as visible mucus layer was present and averaged per mouse, and a fold change compared to an uninfected control average was calculated for each timepoint. *n* = 3-7. (f) 37Total fluorescence was calculated and normalized to tissue section area to quantify MUC2 protein levels. A fold change compared to an uninfected control average was calculated for each timepoint. *n* = 3-8. (g) *Muc2* gene expression was measured using qPCR and a fold change in mRNA expression was calculated compared to an uninfected control average at each timepoint. *n* = 8. Data are shown as box plots (median and interquartile range) with min/max whiskers. *indicates *p* < 0.05, ** indicates *p* < 0.01, ***indicates *p* < 0.001 (Student’s T-test).

To quantify goblet cells in the intestines of mice infected with *G. muris*, PAS/AB staining was performed to visualize goblet cells, and goblet cells were counted per crypt-villus unit in the jejunum, and per crypt in the colon. At day 2 post-infection (PI), an increase in goblet cell number was observed in the jejunum in *G. muris*-infected animals (Figure 1d,e). Expression of the goblet cell-associated differentiation factor *klf4* was similarly upregulated in the jejunum in infected mice at day 2 PI (Figure 1f). No differences in either goblet cell number (Figure 1g,h) or *klf4* expression (Figure 1i) were observed in the jejunum at day 7 or day 30 PI, or at any timepoint in the colon. *Muc2* gene expression was measured using quantitative PCR (qPCR). In the jejunum in *G. muris* -infected mice, *Muc2* mRNA levels were downregulated at day 2, day 7, and day 30 PI (Figure 2c). In the colon, *Muc2* expression was significantly downregulated only at day 30 PI (Figure 2g). To determine whether changes to gene expression corresponded to changes in MUC2 protein levels, tissues were stained with a MUC2 specific antibody. MUC2 protein levels were not found to be significantly altered upon *G. muris* infection in either the jejunum or the colon at any timepoint (Figure 2a–f). However, significant thinning of the colonic mucus gel layer was observed in *G. muris* -infected mice at day 2 PI (Figure 2e), despite both *Muc2* mRNA and MUC2 protein levels remaining similar between groups at this timepoint (Figure 2f,g).

### *Core synthase expression is altered in mucus of* Giardia *infected mice*

**Figure 3.**
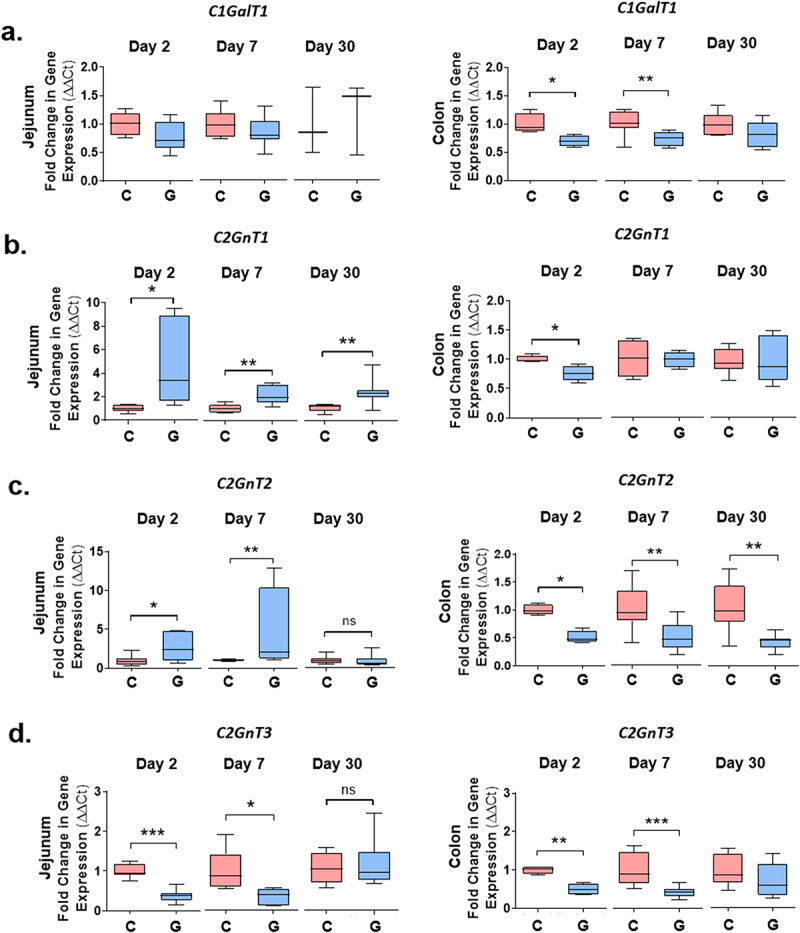
Core synthase expression is altered upon *Giardia* infection in mouse jejunum and colon. Expression of the core synthase genes (a) *C1GalT1*, (b) *C2GnT1*, (c) *C2GnT2*, and (d) *C2GnT3* was measured in jejunum and colon tissues from C57BL/6 mice infected with *Giardia muris* trophozoites for 2, 7, or 30 days using qPCR. A fold change in mRNA levels was calculated compared to an uninfected control at each timepoint. Data are shown as box plots (median and interquartile range) with min/max whiskers. *p < 0.05, **p < 0.01, ***p < 0.001 (student’s T-test). *n* = 4-8.

The backbone of a mucin *O*-linked glycan typically consists of repeating units of Gal, GalNAc, and GlcNAc built upon 1 of 4 possible core structures. To determine whether the abundance of these sugars was altered in response to *G. muris* infection, tissues were stained with fluorescein-coupled lectins, and RT-qPCR was performed to measure changes in the expression of core synthase enzymes. At day 2 and day 7 PI, expression of the core 2 synthases *C2GnT1* and *C2GnT2* was significantly upregulated in the jejunum in *G. muris* infected mice compared to uninfected controls (Figure 3b,c). Expression of another core 2 synthase, *C2GnT3*, was significantly downregulated at the same timepoints (Figure 3d). At day 30 PI, expression of *C2GnT2* and *C2GnT3* had returned to control levels, while *C2GnT1* expression remained elevated in infected mice (Figure 3b–d). In contrast, in the colon, the expression of all four core synthase enzymes was downregulated at day 2 PI (Figure 3, a–d). At day 7 PI, *C1GalT1*, *C2GnT2*, and *C2GnT3* expression remained significantly reduced in infected mice, and at day 30 PI, only *C2GnT2* expression was reduced in infected mice (Figure 3a–d). Results were similar when mice were infected with *G. duodenalis* isolate GS/M, where expression of *C2GnT1* and *C2GnT2* was elevated, and expression of *C2GnT3* reduced in the jejunum in comparison to controls at day 7 PI (Sup. Figure S2). Expression of *C2GnT2* and *C2GnT3* was similarly reduced in the colon in both *G. muris* and *G. duodenalis* infected mice in comparison to controls (Figure 3, Sup Figure S2). In contrast, *C1GalT1* expression was reduced in the colon at day 7 PI only in response to *G. muris* infection, while *C2GnT1* expression was significantly upregulated in response to *G. duodenalis* infection, but remained unchanged in response to *G. muris* infection (Figure 3, Sup Figure S2). *C3GnT* expression was not detectable using qPCR in either the jejunum or distal colon.

The fluorescein-coupled lectins DBA, WGA, and PNA were used to detect GalNAc, GlcNAc, and Gal, respectively (Sup Figure S3). Despite changes in the expression of core synthase genes, no significant alterations in the abundance of these sugars were detected upon *G. muris* infection in either the jejunum or the colon at any timepoint. In *G. duodenalis* infected mice, only GalNAc showed a statistically significant increase in abundance in the jejunum of *G. duodenalis* infected mice (Sup. Figure S2C, S3).

### *Sulfomucin and sialomucin abundance is altered upon* Giardia *infection*

**Figure 4.**
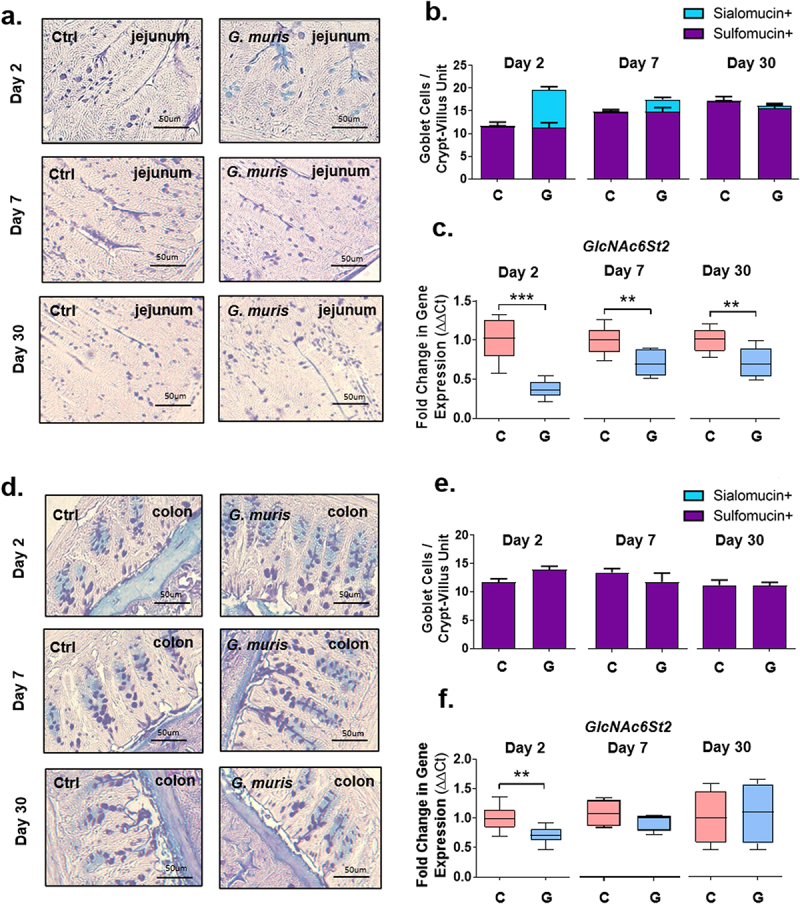
*Giardia* infection alters mucin sulfation and sulfotransferase gene expression in mouse intestines. Jejunum (a) and colon (d) sections from C57BL/6 mice infected with *Giardia muris* trophozoites for 2, 7, and 30 days were stained with high iron diamine/alcian blue staining. Sulfomucin positive (purple) and sialomucin positive (blue) goblet cells were counted per crypt-villus unit in the jejunum (*n* = 8) (b) and per crypt in the colon (*n* = 4-5) (e). Expression of the mucin-associated sulfotransferase GlcNAc6St2 was measured in (c) the jejunum (*n* = 8) and (f) the colon (*n* = 4-8), using qPCR, and a fold change was calculated compared to the uninfected control average for each timepoint. qPCR data is shown as box plots (median and interquartile range) with min/max whiskers. HID/AB staining is shown as bar graphs with error bars indicating SEM. *p < 0.05, **p < 0.01, ***p < 0.001 (student’s T-test).

Sulfation and sialylation are common terminal modifications for mucin *O*-linked glycans in the murine's small and large intestines. To determine whether terminal modification of mucin *O*-glycans was altered upon *Giardia* infection, HID/AB staining was performed to differentiate between goblet cells predominantly containing sulfomucins (Hid+) versus those predominantly containing sialomucins (Hid-). In control mice, goblet cells in the jejunum and colon were predominantly Hid+, indicating the presence of sulphomucins (Figure 4a,d). Upon *G. muris* infection, however, goblet cell hyperplasia as well as the appearance of significant numbers of Hid-goblet cells was observed in the jejunum at day 2 PI, and to a lesser extent at day 7 PI (Figure 4a,b), suggesting an overall reduction in sulphomucin abundance. Most Hid- goblet cells were observed in the small intestinal crypts or near the base of the villi. No alterations to goblet cell number or staining pattern were observed at any timepoint in the colon (Figure 4d,e). The predominant type of sulfate modification in murine intestinal mucins is GlcNAc-6-*O* sulfation, mediated by a single enzyme, the sulfotransferase GlcNAc6ST2, encoded by the *Chst4* gene.^19^ Expression of *GlcNAc6St2* was downregulated in response to *G. muris* infection in the jejunum at all three timepoints, with the most significant downregulation occurring at day 2 PI (Figure 4c). In addition, *GlcNAc6ST2* mRNA levels were reduced in the colons of infected mice at day 2 PI but were similar between groups at day 7 and day 30 (Figure 4f).

**Figure 5.**
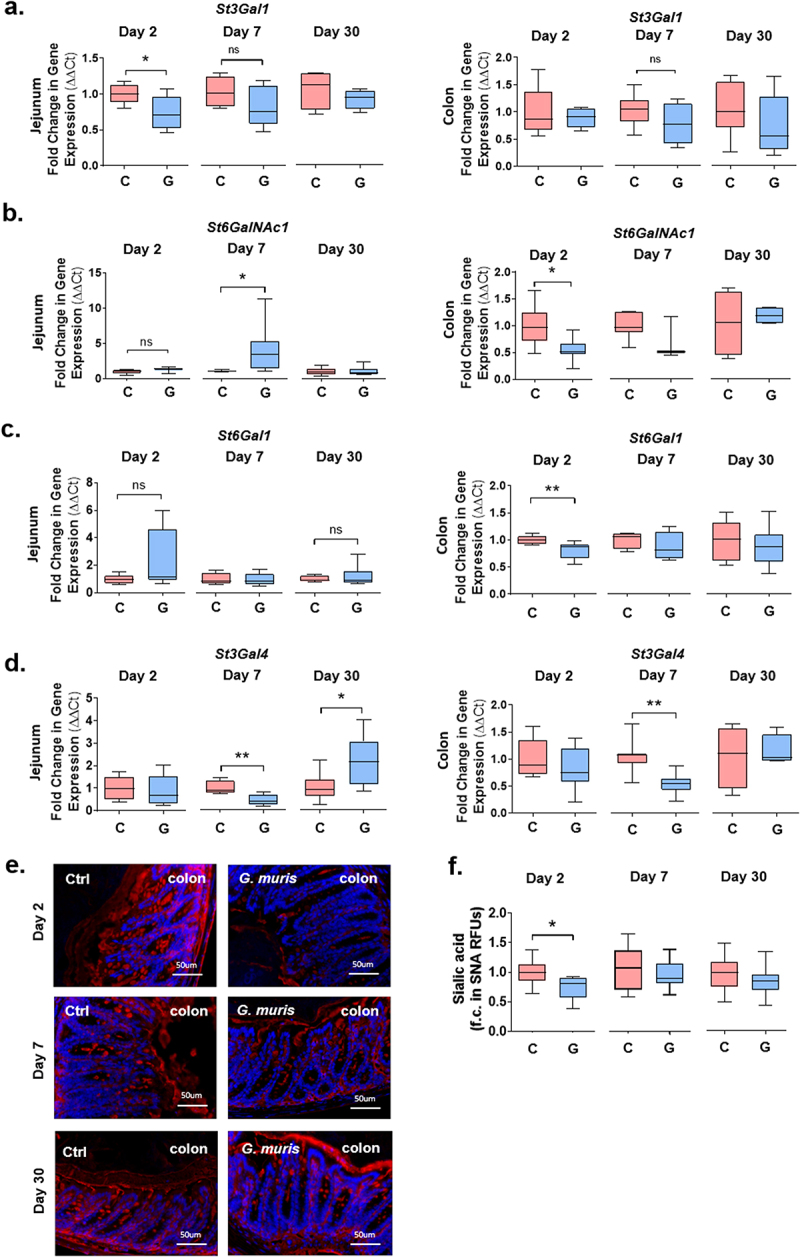
*Giardia* infection alters mucin sialylation and sialyltransferase gene expression in the mouse colon. (a) Expression of the mucin-associated sialyltransferases (c) *St3Gal1*, (d) *St6GalNAc1*, (e) *St6Gal1* and (f) *St3Gal4* was measured in colon at day 2, 7, and 30 PI using qPCR, and a fold change in mRNA expression was calculated compared to an uninfected control average. *n* = 4-8. (e) Colon sections from C57BL/6 mice infected with *Giardia muris* trophozoites for 2, 7, and 30 days were stained with a fluorescein-coupled SNA to detect 38 sialic acid and (f) total fluorescence was calculated and normalized to tissue section area to quantify sialic acid levels. A fold change compared to an uninfected control average was calculated for each timepoint. *n* = 8. Data are shown as box plots (median and interquartile range) with min/max whiskers. *p < 0.05, **p < 0.01 (student’s T-test).

Expression of several sialyltransferases known to be expressed in the mouse GI tract was measured using qPCR. In the jejunum at day 2 PI, *St3Gal1* expression was significantly reduced in *G. muris* infected mice in comparison to control mice (Figure 5a). At day 7 PI, expression of *St6GalNAc1* increased significantly upon infection (Figure 5b), while *St3Gal4* expression decreased (Figure 5d). At day 30 PI, the expression of *St3Gal4* was upregulated in *Giardia* infected mice in comparison to controls (Figure 5d). In the colon, the expression of the sialyltransferases *St6GalNAc1* and *St6Gal1* was significantly decreased at day 2 PI (Figure 5b,c). Similarly, at day 7 PI, infection caused a significant decrease of *St3Gal4*, while *St6GalNAc1, St6Gal1*, and *St3Gal1* showed a trend toward reduction (Figure 5a–d). By day 30, no significant alterations in gene expression were observed between the groups. In *G. duodenalis* infected mice, a similar decrease in *GlcNAc6St2* and *St3Gal4* mRNA expression was observed in the jejunum at day 7 PI, while no alterations to gene expression were observed in the colon at day 7 PI in contrast to *G. muris* infected mice (Sup. Figure 2).

To detect the sialic acid abundance, tissues were stained with fluorescein-coupled SNA. While no specific signal was detectable in the jejunum in this study (data not shown), a significant decrease in SNA staining was observed in the colon in response to *G. muris* infection at day 2 PI, while at day 7 and day 30 PI, sialic acid levels were similar between groups (Figure 5e,f).

### *Jejunal fucose abundance is altered in* Giardia-*infected mice*

**Figure 6.**
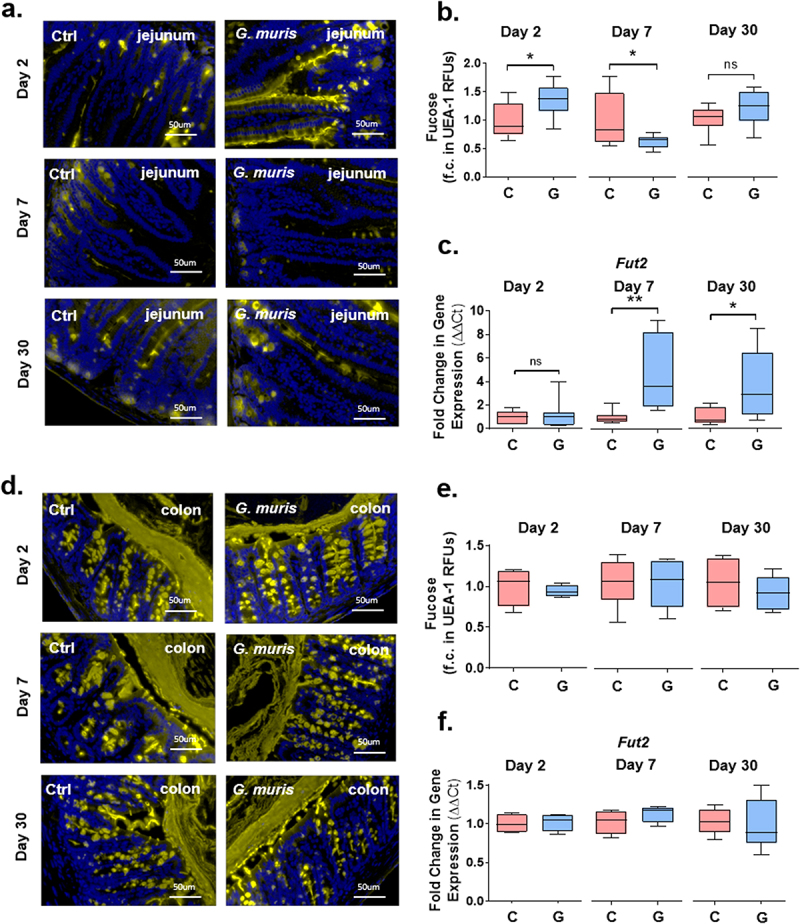
*Giardia* infection alters fucose abundance and fucosyltransferase-2 gene expression in mouse jejunum. Jejunum (a) and colon (d) sections from C57BL/6 mice infected with *Giardia muris* for 2, 7, or 30 days were stained with fluorescein-coupled UEA-1 to detect fucose. Total fluorescence was calculated and normalized to tissue section area to quantify fucose levels in the jejunum (b) or colon (e). A fold change compared to an uninfected control average was calculated for each timepoint (*n* = 8). Expression of fucosyltransferase-2 (*Fut2*) was measured in (c) the jejunum and (f) the colon using qPCR, and a fold change in mRNA expression was calculated compared to an uninfected control average (*n* = 8). Data are shown as box plots (median and interquartile range) with min/max whiskers. *p < 0.05, **p < 0.01 (Student’s T-test).

Fucosylation is another common type of terminal modification for intestinal mucin *O*-glycans, and the abundance of fucosylated mucins is frequently altered during dysbiosis and inflammation. To measure the fucose abundance in *Giardia* infected mice, tissues were stained with fluorescein-coupled UEA-1 to detect α-1,2 linked fucose. In the jejunum in *G. muris* infected mice, the UEA-1 signal was increased at day 2 PI but decreased at day 7 PI compared to uninfected controls (Figure 6a,b). The expression of fucosyltransferase-2 (*Fut2*), the fucosyltransferase responsible for most mucin glycan fucosylation, was significantly increased in the jejunum in infected mice at both day 7 and day 30 PI (Figure 6c). No changes to either the fucose abundance or *Fut2* gene expression were observed at any timepoint in the colon (Figure 6 d–f). Similarly, in *G. duodenalis*-infected mice, a decrease in UEA-1 staining was observed in the jejunum at day 7 PI, concomitant with increased expression of *Fut2* mRNA in comparison to uninfected controls (Sup. Figure S2).

### *The acute phase of* G. muris *infection is associated with microbiota dysbiosis and increased cecal SCFA levels*

**Figure 7.**
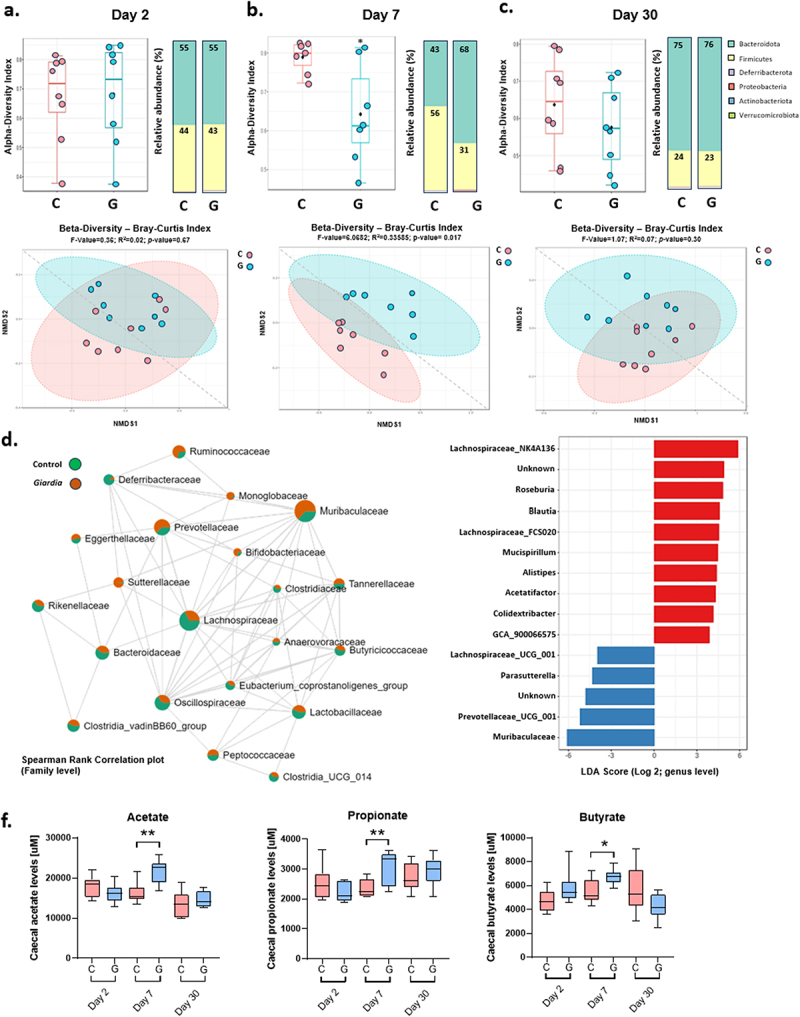
*Giardia* infection alters fecal microbiota composition. 16S sequencing was performed on fecal DNA from uninfected and *Giardia muris* infected mice. Alpha and beta diversity index and the relative abundance of major bacterial phyla at day 2 (a), day 7 (b), and day 30 (c) post-infection (*n* = 8). (d) Spearman rank correlation plot in uninfected and G. muris infected mice at day 7 post-infection. (e) LDA scores at the genus level for control versus G. muris infected mice at day 7 PI. (f) Short-chain fatty acid abundance in mouse cecum at day 2, day 7, and day 30 post-infection (*n* = 8). SCFA quantifications are shown as box plots (median and interquartile range) with min/max whiskers. *p < 0.05, **p < 0.01 (student’s T-test).

*G. muris-*infected mice exhibited significant alterations to the fecal microbiota composition at the phylum level at the peak of infection (day 7), with a decrease of Firmicutes and an increase in the relative abundance of Bacteroidota compared with uninfected mice (Figure 7b). The β-diversity metric (Bray-Curtis index) showed significant dissimilarity between Control and *G. muris* infected mice at day 7 (F-value = 6.0682; R_2_ = 0.33585; p-value = 0.017) (Figure 7b), but not at day 2 (F-value = 0.36; R_2_ = 0.002; p-value = 0.67) or day 30 PI (F-value = 1.07; R_2_ = 0.07; p-value = 0.30) (Figure 7a and b). The α-diversity metric (Simpson’s diversity index) was significantly decreased in *G. muris* infected mice at day 7 indicating a loss in bacterial diversity (richness and evenness) during the acute phase of infection (Figure 7b). No significant changes in α-diversity metric were observed at the early (day 2) and clearance stages (day 30) of infection (Figure 7a and c). Linear Discriminant Analysis (LDA) and Spearman correlation analysis showed that differences between Control and *G. muris*-infected mice at day 7 (peak of infection) were largely driven by microbial shifts in *Lachnospiraceae*, *Rikenellaceae* (*e.g*., *Alistipes*), *Deferribacteraceae*, and *Bacteroidaceae*, which were significantly decreased in *Giardia*-infected mice (LDA score > 3, *p* < 0.05), while members of *Muribaculaceae*, *Prevotellaceae*, and Betaproteobacteria (e.g. *Parasutterella*) were significantly increased upon infection (LDA score < −3, *p* < 0.05) (Figure 7d). The production of Short-Chain Fatty Acids (SCFAs) reflects glycan fermentation and is often altered in response to intestinal infection. Acetate, butyrate, isobutyrate, propionate, valerate, and isovalerate concentrations were quantified in the cecal contents of mice infected with *G. muris* for 2, 7, and 30 days. The cecal concentrations of acetate, propionate, and butyrate were modestly but significantly increased at day 7 PI in response to *G. muris* infection (Figure 7f). No alterations to SCFA production were detected at day 2 or day 30 PI (Figure 7f).

## Alterations to mucin glycosylation are largely microbiota-dependent

**Figure 8.**
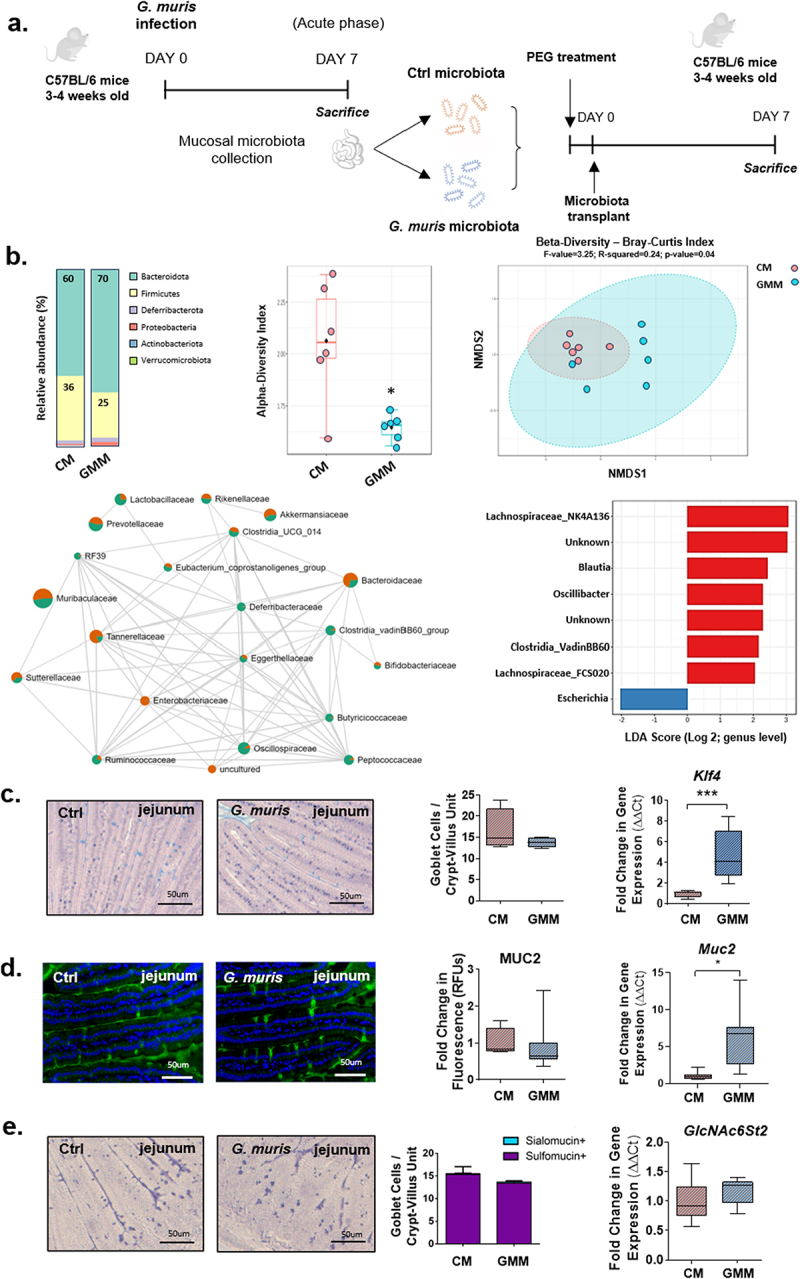
Expression of goblet cell-associated genes is altered in mice transplanted with a *Giardia*-modified small intestinal microbiota. (a) Experimental design for small intestinal microbiota transplant. 3-4-week-old C57BL/6 mice were treated with polyethylene glycol to deplete the intestinal microbiota. Mice were transplanted with small intestinal bacteria isolated from uninfected or *Giardia muris* infected mice. (b) 16S microbiota characterization in fecal DNA from microbiota transplant recipient mice (*n* = 6). (c) Jejunum sections from control microbiota and *G. muris* modified microbiota recipient mice were stained with period acid/alcian blue staining, and goblet cells were counted per crypt-villus unit in the 39jejunum (*n* = 4). Klf4 gene expression was measured using qPCR (*n* = 4-8). (d) Jejunum sections were stained with a MUC2 antibody and fluorescence was quantified and normalized to total tissue section area as a measure of total MUC2 protein levels. A fold change was calculated compared to the control microbiota recipient average (*n* = 5-8). Muc2 gene expression was measured using qPCR (*n* = 8). (e) High iron diamine (Hid)/alcian blue staining was performed and sialomucin positive (blue) and sulfomucin positive (purple) goblet cells were counted per crypt-villus unit (*n* = 8). Expression of the mucin-associated sulfotransferase GlcNAc6St2 was measured using qPCR (*n* = 4-8). Gene expression and PAS/AB staining quantifications are shown as box plots (median and interquartile range) with min/max whiskers. HID/AB staining is shown as a bar graph with error bars indicating SEM. *indicates *p* < 0.05. **indicates *p* < 0.01 (student’s T-test).

Mucosal glycosylation patterns and microbiota composition and function are closely linked.^1,2,4^ To assess the role of *Giardia*-induced dysbiosis in the observed changes in mucin *O*-glycosylation patterns and glycosyltransferase gene expression, a small intestinal microbiota transplant was performed. Small intestinal bacteria were collected from uninfected and *G. muris* infected mice at day 7 PI and transplanted into mice treated with PEG to deplete the native intestinal microbiota (Figure 8a). As observed for *G. muris*-infected mice compared to controls, no changes in mouse weight gain, intestinal transit, or fecal water weight were observed at day 7 post microbiota transplant in mice that received a *G. muris*-modified microbiota compared to those that received a control microbiota (Sup Figure 4a,b and c).

Mice transplanted with a *G. muris*-modified microbiota (GMM) exhibited an alteration of fecal microbiota composition at the phylum level at day 7 post-transfer, with a decrease of Firmicutes and an increase of Bacteroidota compared with mice that received a control microbiota (CM) (Figure 8b). The β-diversity metric (Bray-Curtis index) showed significant dissimilarity between CM and GMM groups at day 7 post-transplant (F-value = 3.25; R^2^ = 0.24; p-value = 0.04). The α-diversity metric (Shannon’s diversity index) was significantly decreased in the GMM group compared to CM mice, indicating a loss in bacterial diversity similar to that seen in *G. muris* infected mice. Linear Discriminant Analysis (LDA) and Spearman correlation analysis showed differences between CM and GMM microbiota characterized by microbial shifts in *Lachnospiraceae*, *Blautia*, *and Clostridia*, which were significantly decreased in GMM mice (LDA score > 2, *p* < 0.05). Interestingly, *Enterobacteriaceae* (*e.g. Escherichia*) were significantly increased in GMM mice compared with CM mice (LDA score < −2, *p* < 0.05) (Figure 8b). The cecal production of SCFAs was not different between CM and GMM mice (Sup. Figure S4).

**Figure 9.**
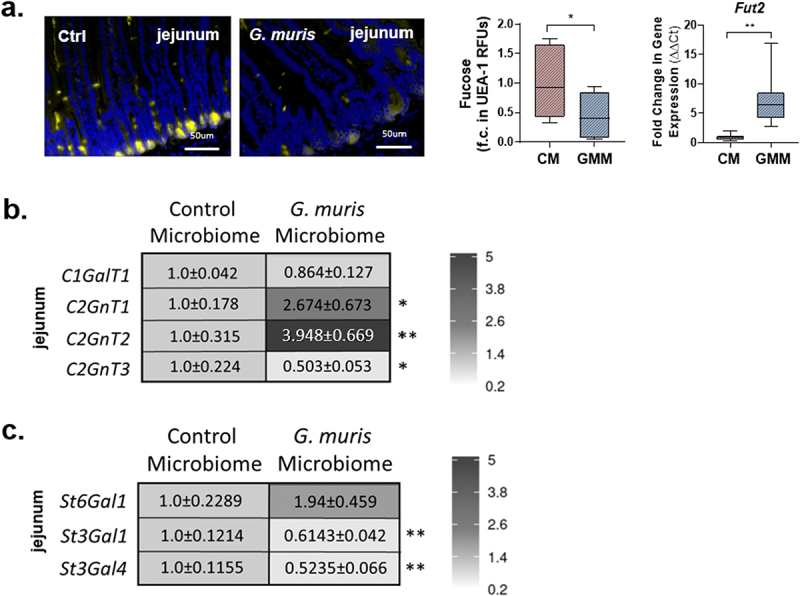
Glycan abundance and glycosyltransferase gene expression is altered in *Giardia* modified microbiota transplant recipient mice. (A) Jejunum and colon sections from C57BL/6 mice transplanted with control or *Giardia muris* modified small intestinal murine bacteria were stained with UEA-1 to detect fucose. Fluorescence was calculated and normalized to tissue area, and a fold change in fluorescence was calculated compared to the control microbiota recipient average (*n* = 4-8). Gene expression of (A) fucosyltransferase-2, the mucin-associated core synthases (B) C1GalT1, C2GnT1, C2GnT2, and C2GnT3, and (C) the mucin-associated sialyltransferases St6Gal1, St3Gal1, and St3Gal4 were measured in the jejunum using quantitative PCR. A fold change was calculated compared to the uninfected control average for each timepoint (*n* = 4-8). Data are shown as box plots (median and interquartile range) with min/max whiskers. * indicates *p* < 0.05. **indicates *p* < 0.01. ***indicates *p* < 0.001 (student’s T-test).

No changes in goblet cell number or MUC2 protein abundance were observed between the groups (Figure 8c,d), although both *Klf4* and *Muc2* gene expression were found to be significantly increased in the jejunum in GMM mice compared to CM mice (Figure 8c,d). As was observed in *G. muris* infected mice at day 7 PI, the abundance of sulfomucin and sialomucin positive goblet cells did not differ significantly between CM and GMM mice at day 7 post-transplant (Figure 8e). In contrast, however, while *G. muris*-infected mice showed downregulation of *GlcNAc6ST2* gene expression in the jejunum at all three timepoints studied, no downregulation of this gene was observed in GMM mice compared to CM mice (Figure 8e). Sialic acid abundance was similar between CM and GMM mice in the colon (Sup. Figure S5), while both *St3Gal1* and *St3Gal4* expression were decreased in the jejunum, and *St6Gal1* and *St3Gal4* expression were decreased in the colons of GMM mice (Figure 9c and Sup Figure S5). Fucose abundance was significantly reduced in the jejunum in GMM mice compared to CM mice, while *Fut2* gene expression was significantly increased (Figure 9a and b). No alterations to fucose abundance or *Fut2* expression were observed in the colon in either study (Figure 9a and b, Sup Figure S5).

Expression of core synthase genes was found to be similar between studies, as in both *G. muris* infected mice and in GMM mice, *C2GnT1* and *C2GnT2* expression were significantly elevated, while *C2GnT3* was significantly reduced in the jejunum in comparison to the respective controls (Figure 9b). In the colon, *C2GnT2* and *C2GnT3* expression was significantly downregulated in both *G. muris* infected and GMM mice, while *C1GalT1* was downregulated only in *G. muris*-infected mice and was similar between CM and GMM mice (Figure 9b). *C2GnT1* expression did not vary at day 7 in either study (Figure 9b). As was observed in *G. muris* infected mice, no alterations to GalNAc abundance were detected in either the jejunum or colon in GMM mice compared to CM mice (Sup. Figure S6).

To determine whether alterations to glycosyltransferase gene expression may be due in part to direct interactions with trophozoites, the human colonic mucus-producing cell line LS174T was infected with *G. duodenalis* isolate NF or isolate GS/M (Sup. Figure S7A and B). After 3 hours of infection, no significant differences in glycosyltransferase gene expression were observed in cells infected with either isolate, suggesting that *Giardia* alone does not induce direct changes to mucin-associated glycosyltransferases in goblet cells (Sup. Figure S6A and B).

## Discussion

Disruption of the intestinal mucus barrier has been observed in a broad range of intestinal diseases, including bacterial and parasitic infections and IBD.^4,20–23^ *Giardia* was previously found to disrupt the mucus barrier by hyperactivating goblet cell secretion and by degrading mucins with its secreted cysteine proteases.^11,12^ The present study reveals that, in addition to altering the production and secretion of mucins, *Giardia* alters mucin biochemistry by disrupting *O*-glycosylation patterns in a manner dependent in part upon a dysbiotic microbiota. Abnormal mucin glycosylation has previously been associated with altered mucus barrier function and dysbiosis,^1,2,5^ suggesting that this novel mechanism of pathogenesis may contribute to several key aspects of *Giardia* pathogenesis.

Using murine models of infection with either *G. muris* or *G. duodenalis*, the present findings reveal that *Giardia* infection can alter mucin glycosylation and the expression of mucin-associated glycosyltransferases in both the small intestines, at the site of parasite colonization, and in the distal colon, where *Giardia* trophozoites are absent. These abnormalities were observed as early as day 2 post-infection, and many persisted until day 30 post-infection, at which time the majority (6 of 8) of *G. muris-*infected mice had an undetectable parasite burden. The finding that *Giardia* can induce alterations to mucin glycosylation beyond the site of infection and that these alterations can persist after parasite clearance, is of particular interest in the context of post-infectious and extra-intestinal complications, such as post-infectious irritable bowel syndrome (IBS).^10^ IBS has been associated with intestinal barrier dysfunction,^24^ resulting in visceral pain and diarrhea. Further research is required to determine whether and how mucin glycosylation patterns may be disrupted in patients with IBS, and whether *Giardia*-induced glycan alterations may be responsible for increased intestinal permeability that may contribute to post-infectious disease manifestations.

At day 2 PI, increased goblet cell abundance and increased expression of the goblet cell-associated differentiation factor *Klf4* were observed in the jejunum in *G. muris*-infected mice. Despite a significant downregulation of MUC2 mucin gene expression in the jejunum at day 2, day 7, and day 30 PI, no differences in MUC2 protein could be detected via immunohistochemistry. Goblet cell hyperplasia is a common and nonspecific host defense mechanism against intestinal parasites and is often accompanied by increased production and secretion of mucus, which helps flush out the pathogen.^25,26^ Downregulation of *Muc2* gene expression may therefore represent a mechanism for *Giardia* to evade this host defense system, as it prevents increased production of MUC2 despite an overall increase in goblet cell number. A previous study similarly observed *Muc2* downregulation at day 7 PI in *G. duodenalis*-infected mouse small intestines^11^ while in the present study, no significant differences in *Muc2* gene expression were observed between uninfected and *G. duodenalis* GS/M infected mice, suggesting that this response may be more transient during infection with *G. duodenalis*, while it is sustained in response to mouse-specific *G. muris*. Consistent with other reports, the present findings show that *G. duodenalis* infections resolved within two weeks^11^, while *G. muris* infections persisted for upward of 3–4 weeks. Further research will be required to determine whether upregulation of mucus production and secretion is involved in *Giardia* clearance, as has been observed in numerous other parasitic infections. Isolate-dependent modulation of mucin gene expression in human cell cultures has previously been found to correlate with isolate-dependent cysteine protease activity.^11,12^

Significant thinning of the colonic mucus gel barrier was observed at day 2 PI. Similar thinning of the colonic mucus barrier has previously been observed in *G. duodenalis*-infected mice at day 7 PI,^11^ and is proposed to contribute to intestinal barrier dysfunction. This was accompanied by significant downregulation in the expression of four core synthase genes, encoding glycosyltransferases responsible for the initial steps in glycan synthesis, in the colons of infected mice at day 2 PI. The observed mucus thinning may therefore be due to reduced mucin glycosylation as a result of the broad downregulation of all major mucin-associated core synthase genes. Loss of core synthase expression in mice has previously been associated with significant barrier dysfunction. In *C1GalT1* deficient mice, loss of core 1 structures causes development of spontaneous colitis,^27,28^ and increased susceptibility to dextran sodium sulfate-induced colitis.^29^ Mucins from mice deficient in *C1GalT1* as well as the core 3 synthase *C3GnT* are more susceptible to proteolysis than mucins from wild-type mucins, indicating an important role for core 1 and core 3-derived glycans for protecting the mucin backbone and maintaining the intestinal mucus barrier.^27^ Mice deficient in *C2GnT2* expression similarly show impaired mucosal barrier function and increased susceptibility to DSS colitis.^30^ Therefore, reduced expression of core synthase genes may result in production of poorly glycosylated mucins, leading to mucus barrier dysfunction during acute *Giardia* infection. No changes in the abundance of Galactose, GalNAc, or GlcNAc were observed at any of the timepoints studied in either the jejunum or colon via staining with fluorescein-coupled lectins. However, lectin staining is not sufficiently specific to detect alterations to individual *O*-glycan structures. More precise and quantitative methods will be required to determine how the observed alterations to core synthase gene expression impact the degree of mucin glycosylation and the length and arrangement of specific *O*-glycan epitopes. It is also possible that Muc2 crosslinking is altered upon *Giardia* infection, which may contribute to mucus barrier thinning. A recent study demonstrated that transglutaminase 3 is critical for colonic mucus barrier function, and the absence of transglutaminase activity in mice was associated with increased degradation of MUC2 and altered mucus properties.^31^ Further research will be necessary to characterize the role of transglutaminases and alterations to Muc2 crosslinking in *Giardia* infection.

In *G. muris* infected mice, we observed a shift in the ratio of goblet cells containing predominantly sialylated or sulfated mucins. In control mice, the vast majority of goblet cells were high-iron diamine (HID) reactive, indicating the presence of sulfated mucins. In *G. muris* infected mice at day 2 PI, and to a lesser extent at day 7 PI, we observed a shift toward increased abundance of non-HID-reactive goblet cells, indicating reduced sulphomucin abundance. In accordance with this observation, *GlcNAc6ST2*, the enzyme responsible for the majority of intestinal mucin *O*-glycan sulfation, was significantly downregulated in *G. muris* infected mice at all three timepoints investigated. Previous studies have demonstrated that sulfomucins play important roles in mucus barrier function, as evidenced by the increased susceptibility of mice deficient in sulfomucin production to infection and inflammation.^19,32^ Further, in mice infected with the parasite *Trichuris muris*, upregulation of sulfomucin production was associated with timely parasite clearance, while mice unable to upregulate sulfomucin production developed chronic infection. Sulfomucins were found to be more resistant to degradation by parasite proteases than sialomucins. Downregulation of sulfomucin production may represent a novel mechanism of *Giardia* pathogenesis that may facilitate disruption of the intestinal mucus barrier by *Giardia* proteases. Cysteine proteases are a key virulence factor for *Giardia* and have previously been shown to be capable of degrading purified human mucins.^11^ Further work will be required to compare the relative susceptibility to degradation by *Giardia* cysteine proteases of mucins with distinct glycosylation profiles.

The expression of mucin-associated sialyltransferases was altered in the small and large intestines in response to *G. muris* and *G. duodenalis* infection. In addition, we observed altered SNA staining patterns in the colon, suggesting alterations to sialic abundance. Alterations were both time dependent and distinct between the small and large intestines. Sialic acid, like sulfate, is a common terminal modification for mucin *O*-glycans, and therefore plays important roles in microbiota selection and metabolism. Alterations to the availability of specific sialylated epitopes may influence microbiota composition by altering binding site and nutrient availability, as CAZymes and binding proteins that target mucin *O*-glycans tend to be highly specific for a limited number of sugar conformations.^5,33,34^

Fucose, another common *O*-glycan terminal modification, was also found to be altered in the small intestines. In the jejunum, expression of the predominantly mucin-associated fucosyltransferase, *Fut2*, was upregulated at both day 7 and day 30 PI in the jejunum. Fucose abundance, detected using the lectin UEA-1, increased at day 2 PI, then decreased at day 7 PI in *G. muris* infected mice compared to controls. No changes were observed in the distal colon. Fucose abundance has previously been linked to microbiota composition. In humans, FUT2 expression controls secretor status, which determines the expression of ABH and Lewis histo-blood group antigens in the intestinal mucosa. Non-secretors have been shown to have a unique microbiota composition, as well as increased risk of developing IBD^35–37^.In addition, *Fut2* knockout mice exhibit a unique microbiota signature, as well as increased susceptibility to DSS colitis.^35,36^ In turn, *Fut2* expression can be regulated by the microbiota, and is induced upon weaning in rodents, as well as upon colonization of germfree or antibiotic treated mice.^38^ It is important to note that this and other glycosyltransferases may be involved in the glycosylation of both secretory mucins and transmembrane mucins, as well as other glycosylated proteins. Further studies will be required to characterize how the observed changes in glycosyltransferase gene expression impact secretory versus cell surface glycoproteins.

*Giardia* infection has previously been associated with alterations to the composition and activity of the microbiota.^39–41^The microbiota has also been found to be a key regulator of mucin glycosylation patterns. We therefore investigated the role of microbiota dysbiosis in *Giardia*-induced alterations to mucin glycosylation. At day 7 PI, we observed a shift in the relative abundance of Firmicutes and Bacteroides and an overall decrease in alpha diversity, in accordance with previous studies in mice^39^ and in humans.^42^ *Giardia* was found to alter relative species abundance at the phylum level, with a decrease in *Lachnospiraceae*, *Rikenellaceae*, *Deferribacteraceae*, and *Bacteroidaceae*, and an increase in *Muribaculaceae*, *Prevotellaceae*, and Betaproteobacteria. The beta diversity metric showed significant dissimilarity between control and *G. muris*-infected mice at day 7 at the peak of infection. The caecal production of acetate, butyrate, and propionate, which are known by-products of glycan fermentation by the anaerobic microbiota, was increased at day 7 PI in response to *G. muris* infection. While members of *Muribaculaceae*,^43^ which were increased in *G. muris*-infected mice, are known producers of propionate, no significant producers of butyrate and acetate were identified. Similarly, we detected alterations to the composition of the small intestinal microbiota isolated for microbiota transplantation in *G. muris* infected mice compared to controls (Sup Figure 8).

Many alterations to glycosyltransferase gene expression observed at the peak of infection (day 7) were also detected in mice transplanted with a *G. muris*-modified microbiota, including altered expression of core synthase genes, sialyltransferase genes, and fucosyltransferase-2. This suggests that at least some of the genes found to be differentially regulated between control and *Giardia*-infected mice are controlled by the small intestinal microbiota. However, several genes did not show microbiota-dependent altered expression in GMM recipient mice, including *GlcNAc6ST2*, *C1GalT1*, *St3Gal1*, and *St6Gal1* depending on the time point of infection and gut region. This suggests that non-microbial factors also regulate glycosyltransferase genes during *Giardia* infection. It is also possible that these genes are regulated by specific bacterial species that were not successfully transplanted in our study. Alternatively, particularly for genes found to be altered in the distal colon, species that do not colonize the small intestine may be involved in gene regulation, as we only collected and transferred the small intestinal microbiota in this study.

At day 7 post-microbiota transplant, the fecal microbiota of GMM recipient mice differed from CM recipient mice, confirmed by a significant β-diversity dissimilarity, decreased α-diversity, and a decrease in Firmicutes abundance, similar to *G. muris*-infected mice. Significant microbial shifts in GMM (e.g., *Lachnospiraceae*, *Blautia*, *Clostridia*) were not associated with a change in known mucus and/or glycan degrading bacterial species. Interestingly, GMM transfer was associated with a significant increase in *Enterobacteriaceae* species, as has been observed in the jejunum in clinical studies.^44^ *Giardia* has previously been shown to disrupt microbiota biofilms and promote the formation of biofilm-dispersed invasive pathobionts.^40^ A recent study showed that the small RNA cargo of extracellular vesicles secreted by *Giardia* actively modifies the growth, behavior, and virulence of *Enterobacteriaceae* species.^45^ The growth and colonization of pathogenic *Enterobacteriaceae* species including *Salmonella* and *Escherichia coli* can also be induced by increased levels of free sialic acid.^46,47^ Whether or not the increased abundance of *Enterobacteriaceae* species may alter enteric mucus glycosylation warrants further research. Further research is required to characterize the role of distal colonic bacteria in the regulation of mucing lycosylation patterns during *Giardia* infection and to determine the contribution of individual bacterial species to the regulation of specific glycosyltransferase genes.

To investigate the role of direct interactions between goblet cells and *Giardia* trophozoites in altered glycosyltransferase gene expression, human mucus-producing cell cultures were infected with *G. duodenalis* NF or *G. duodenalis* GS/M. Neither isolate induced alterations to glycosyltransferase gene expression in human cells, suggesting that altered gene expression is unlikely to be due to direct interactions between parasites and host cells.

In summary, this study characterizes a hitherto unreported mechanism by which an intestinal parasite, *Giardia*, alters mucus biochemical properties throughout the intestinal tract in a manner largely dependent on a dysbiotic microbiota. Findings indicate that alterations to glycosylation patterns and the expression of mucin-associated glycosyltransferases are time dependent, persist even in mice that have cleared the infection, and occur at sites of parasite colonization as well as sites far distal in the intestinal tract, where there is no active infection. This represents a new mechanism by which *Giardia* can disrupt the intestinal mucus barrier. These mechanisms of pathogenesis will be of considerable importance in the study of post-infectious and extra-intestinal complications associated with *Giardia* and other infections.

## Supplementary Material

Supplemental Material

## Data Availability

Data associated with this paper can be found in the Dryad Digital Repository at 10.5061/dryad.05qfttf9p
