# Supplementary material for: Giardia spp.-induced microbiota dysbiosis disrupts intestinal mucin glycosylation
Source: Gut Microbes. 2024 Oct 16;16(1):2412676. doi: 10.1080/19490976.2024.2412676 (PMC11485787; doi:10.1080/19490976.2024.2412676)
Supplement: Supplemental Material [file KGMI_A_2412676_SM4170.zip › Supplementary_Material__17_/Fekete et al 2024_Sup Figures_V2 (1).pdf]

Sup. Figure 1

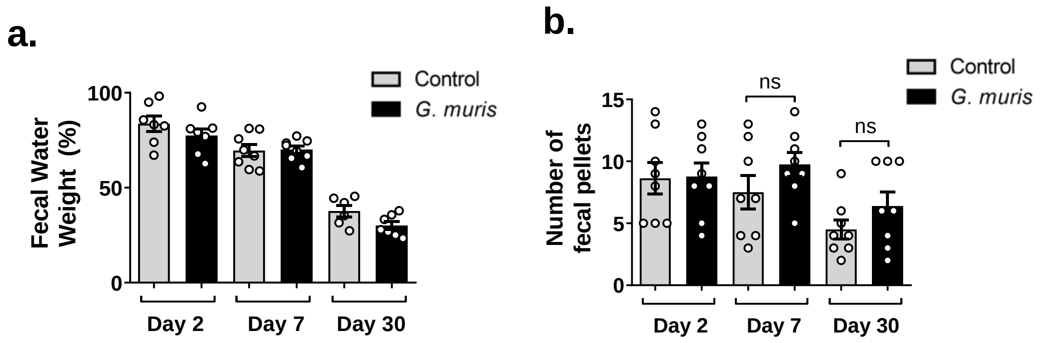
a.
Fecal Water Weight (%)
100
50
0
Day 2
Day 7
Day 30
Control
G. muris
b.
Number of fecal pellets
15
10
5
0
ns
ns
Day 2
Day 7
Day 30
Control
G. muris

Sup. Figure 2

a. **Jejunum**

	Uninfected	*G. duodenalis* GS/M Infected	
Muc2	1.0±0.159	1.668±0.2819	
GlcNAc6St2	1.0±0.0889	0.6618±0.06755	**
Fut2	1.0±0.3249	4.001±0.9721	*
St6GalNAc1	1.0±0.2358	1.74±0.3082	
St6Gal1	1.0±0.1202	1.235±0.1855	
St3Gal1	1.0±0.08684	1.055±0.1541	
St3Gal4	1.0±0.1782	0.3113±0.03854	**
B4Galnt2	1.0±0.1096	0.7535±0.05417	
C1GalT1	1.0±0.06771	0.9329±0.05417	
C2GnT1	1.0±0.09906	2.898±0.4845	***
C2GnT2	1.0±0.235	2.389±0.534	*
C2GnT3	1.0±0.125	0.3312±0.01899	***

b. **Colon**

	Uninfected	*G. duodenalis* GS/M Infected	
Muc2	1.0±0.1294	1.248±0.0783	
GlcNAc6St2	1.0±0.1514	1.051±0.07595	
Fut2	1.0±0.17	1.078±0.04059	
St6GalNAc1	1.0±0.371	0.4227±0.04775	
St6Gal1	1.0±0.1418	1.045±0.2808	
St3Gal1	1.0±0.08287	1.102±0.1576	
St3Gal4	1.0±0.1174	0.8236±0.1053	
B4Galnt2	1.0±0.3048	1.197±0.198	
C1GalT1	1.0±0.1647	1.181±0.2054	
C2GnT1	1.0±0.1262	2.563±0.5717	*
C2GnT2	1.0±0.1341	0.5865±0.0.0924	*
C2GnT3	1.0±0.1149	0.6123±0.09015	*

Values
5
3.75
2.5
1.25
0

c. **Jejunum Day 7 PI**

	Uninfected	*G. duodenalis* Infected	
UEA-1 (Fucose)	1.0±0.3299	0.2296±0.0874	**
DBA (GlcNAc)	1.0±0.0851	0.9783±0.0964	
WGA (GalNAc)	1.0±0.1243	1.48±0.1592	*
PNA (Galactose)	1.0±0.0730	0.9671±0.0939	

Values
1.5
1.125
0.75
0.375
0

Colon Day 7 PI

	Uninfected	*G. muris* Infected
UEA-1 (Fucose)	1.0±0.1103	0.9945±0.14
DBA (GlcNAc)	1.0±0.0654	0.91±0.0996
WGA (GalNAc)	1.0±0.1189	1.079±0.1241
PNA (Galactose)	1.0±0.2226	0.8642±0.1637
SNA (Sialic Acid)	1.0±0.1615	1.392±0.117

Values
1.5
1.125
0.75
0.375
0

Sup. Figure 3

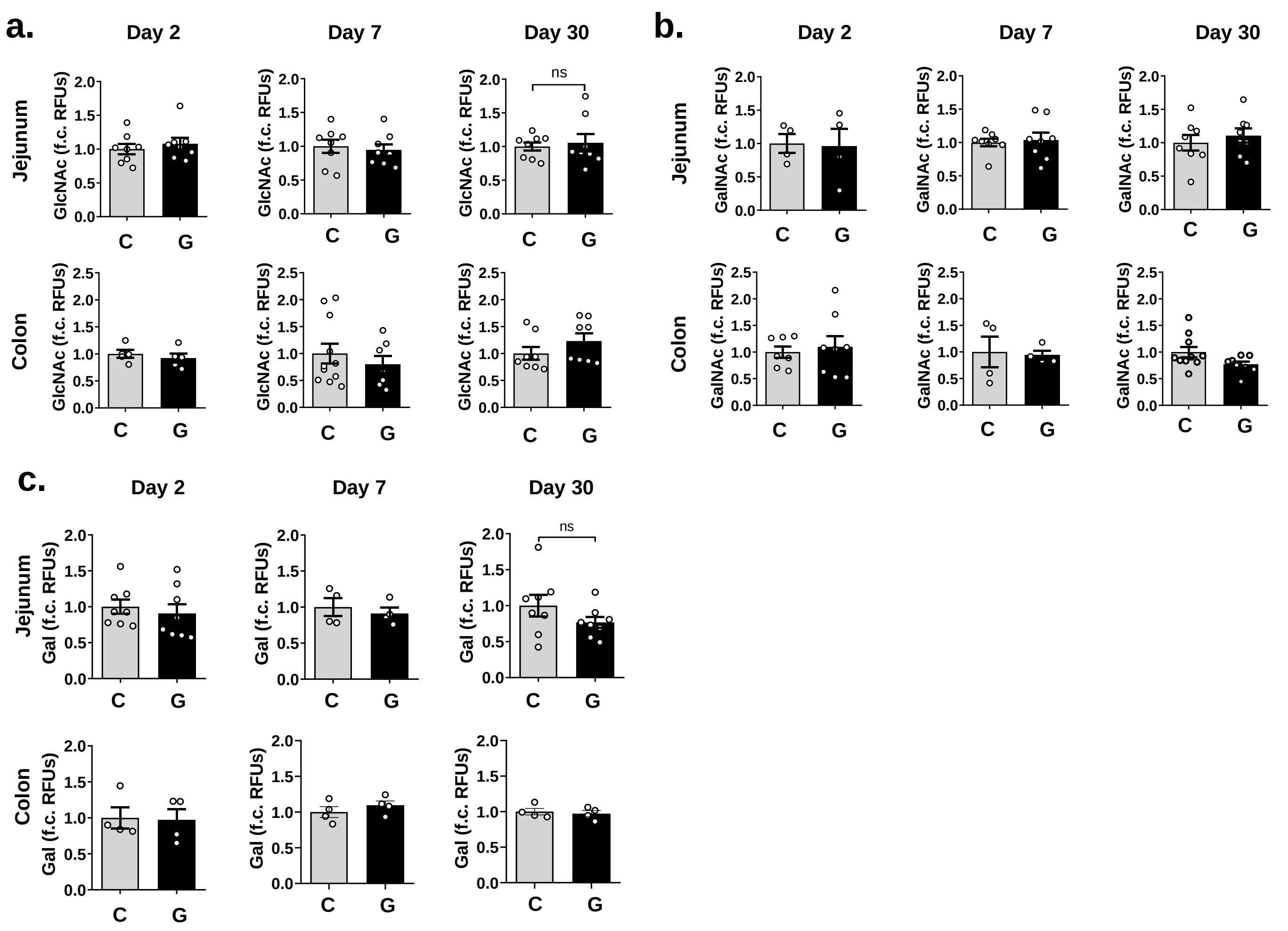
a.
Day 2
Day 7
Day 30
Jejunum
Colon
GlcNAc (f.c. RFUs)
ns
C
G
b.
Day 2
Day 7
Day 30
Jejunum
Colon
GalNAc (f.c. RFUs)
C
G
c.
Day 2
Day 7
Day 30
Jejunum
Colon
Gal (f.c. RFUs)
ns
C
G

Sup. Figure 4

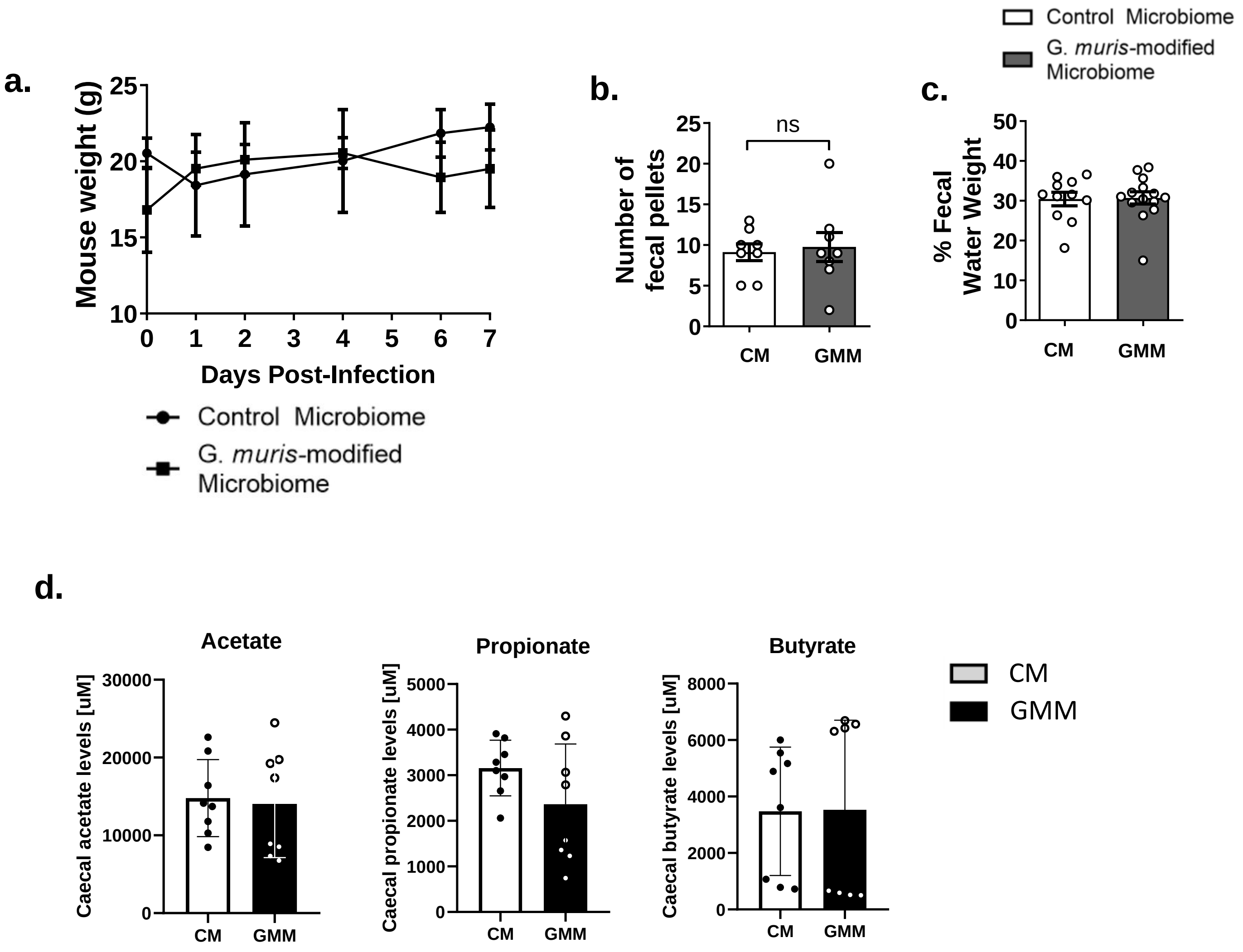

a.
Mouse weight (g)
Days Post-Infection
Control Microbiome
G. muris-modified Microbiome
b.
ns
Number of fecal pellets
CM
GMM
c.
% Fecal Water Weight
CM
GMM
Control Microbiome
G. muris-modified Microbiome
d.
Acetate
Caecal acetate levels [uM]
Propionate
Caecal propionate levels [uM]
Butyrate
Caecal butyrate levels [uM]
CM
GMM

Sup. Figure 5

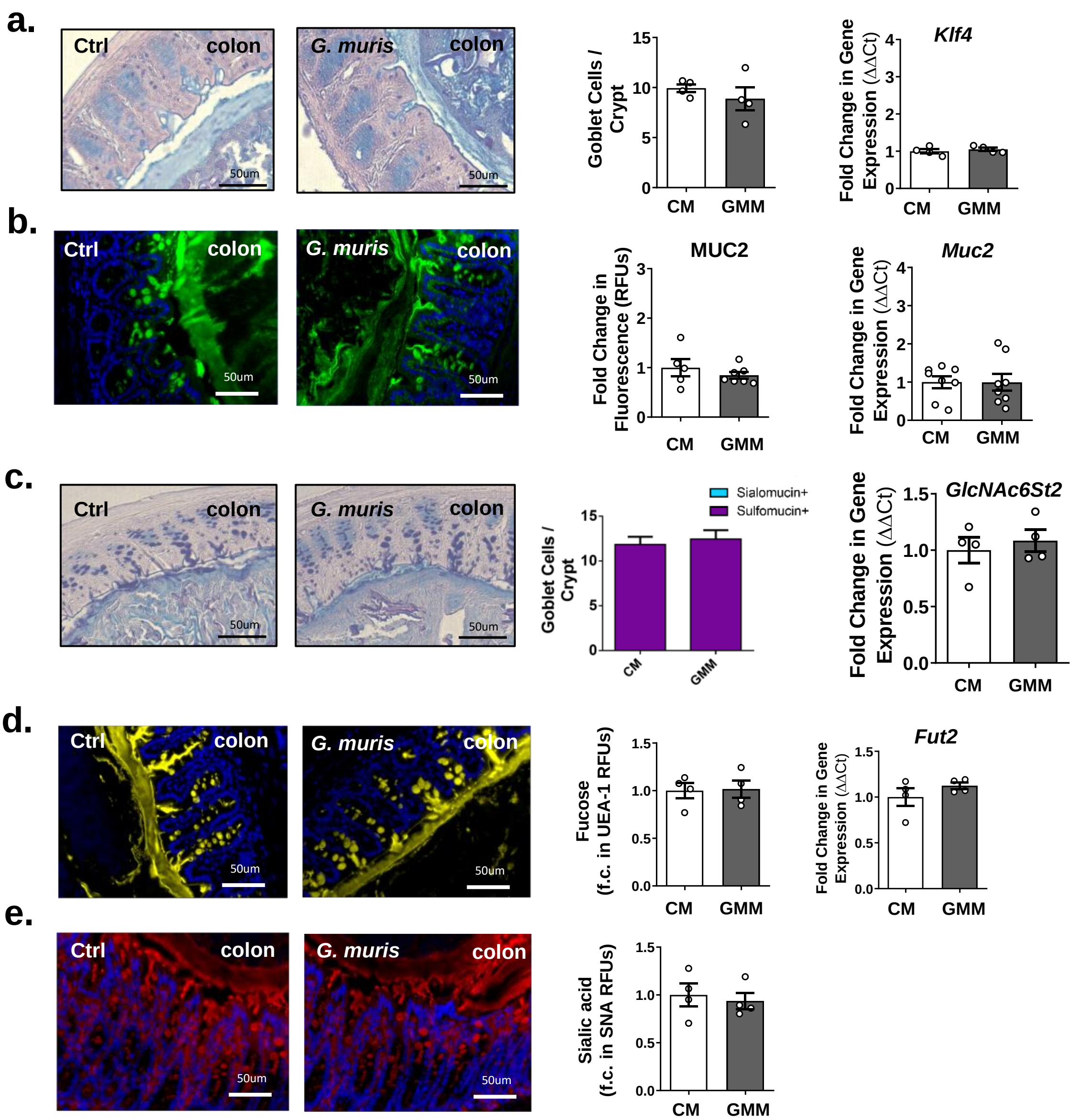

f.

colon

	Control Microbiome	*G. muris* Microbiome	
C1GalT1	1.0±0.0853	0.956±0.046	
C2GnT1	1.0±0.117	0.862±0.090	
C2GnT2	1.0±0.126	0.428±0.044	***
C2GnT3	1.0±0.049	0.722±0.073	**

g.

colon

	Control Microbiome	*G. muris* Microbiome	
St6Gal1	1.0±0.1269	0.5869±0.0543	*
St3Gal1	1.0±0.085	1.055±0.1499	
St3Gal4	1.0±0.1323	0.6207±0.1093	*

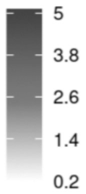

Sup. Figure 6

a.

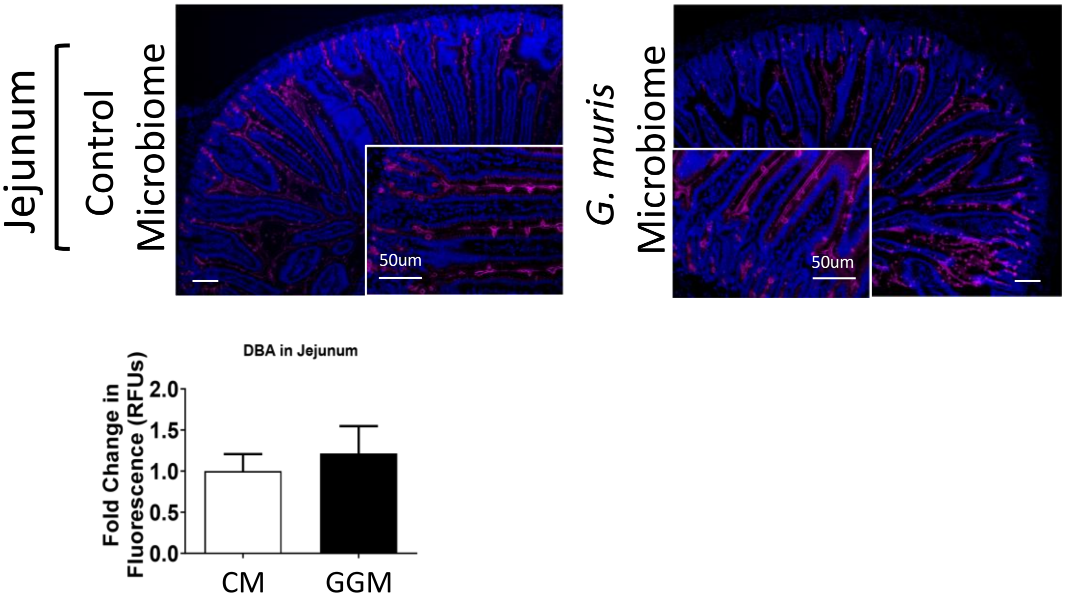

b.

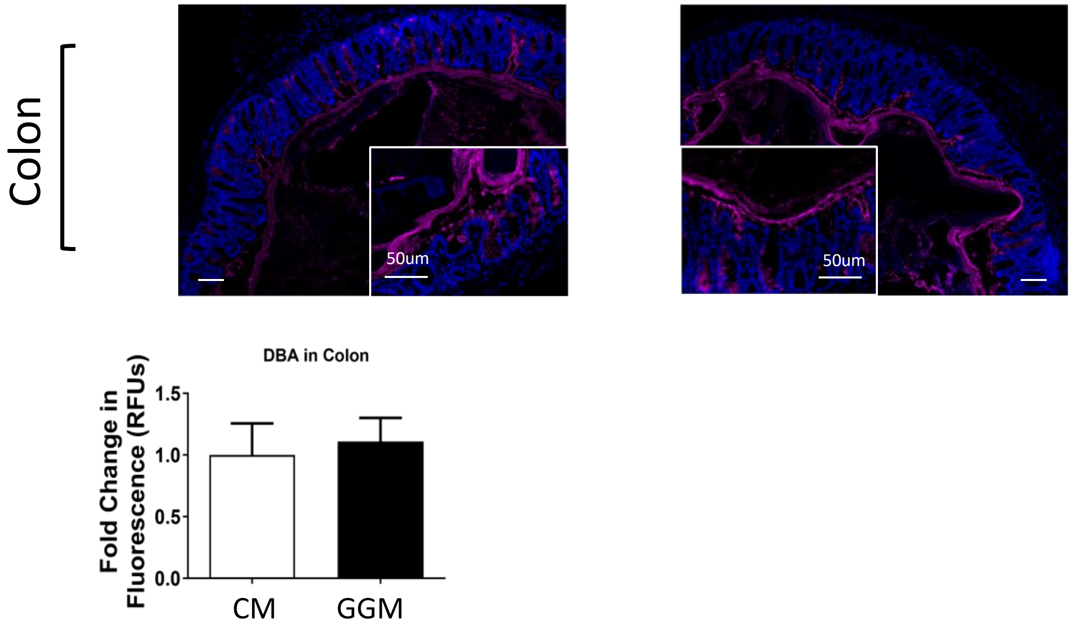

Sup. Figure 7

a.

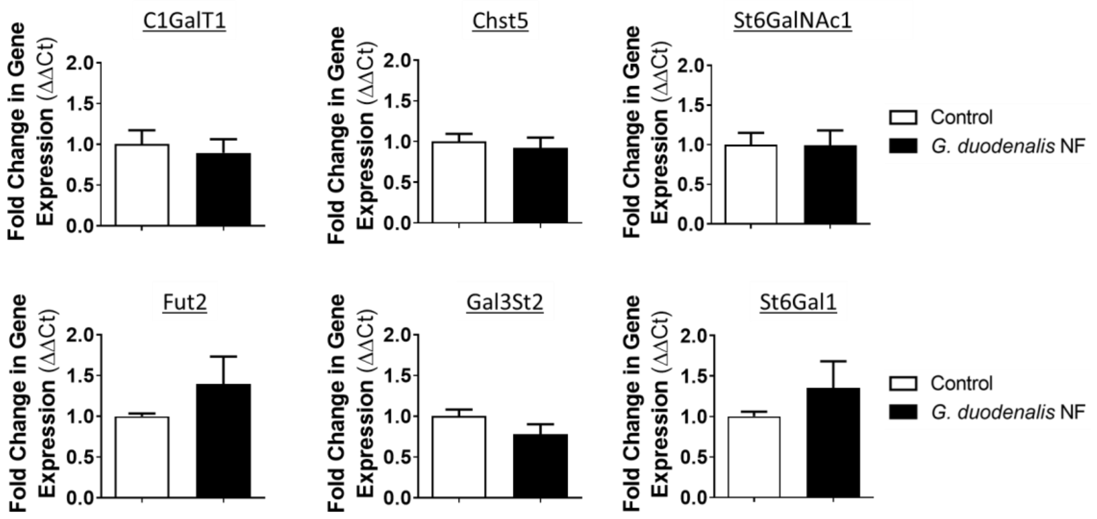

b.

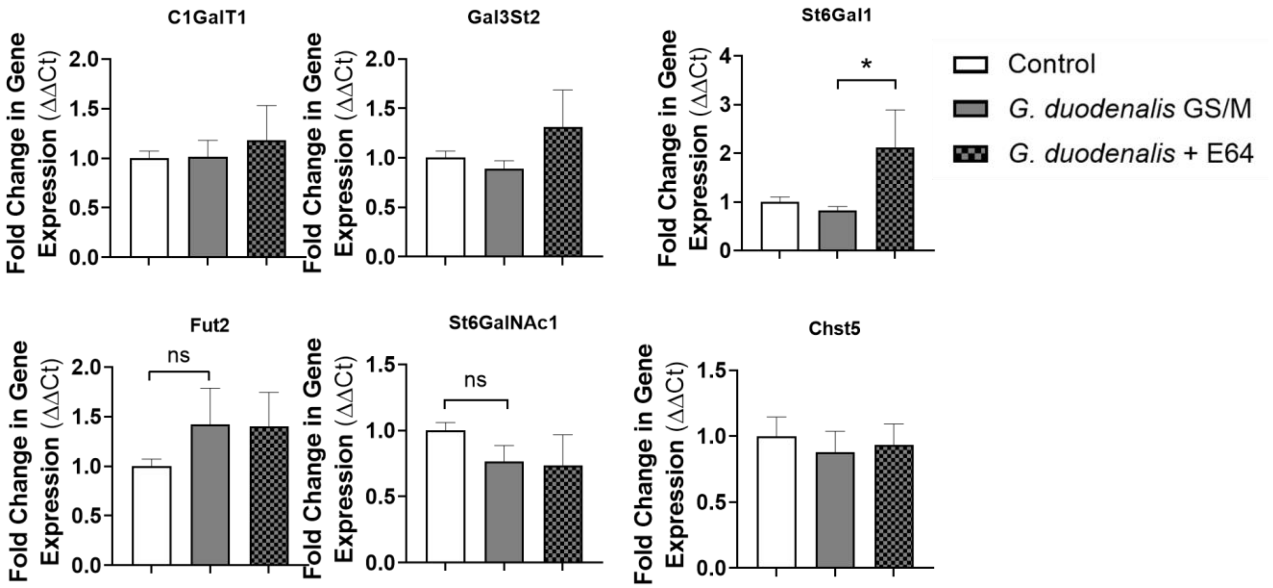

Sup. Figure 8

a

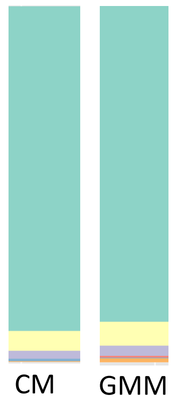

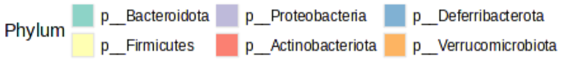

Percent relative abundance (%)

Phulym	CM (stock)	GMM (stock)
Bacteroidota	91.47	88.92
Firmicutes	5.53	6.49
Proteobacteria	2.01	2.70
Deferribacterota	0.63	0.34
Actinobacteriota	0.29	0.68
Verrucomicrobiota	0.07	0.86

b

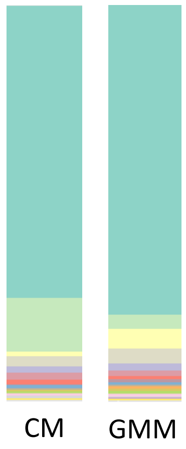

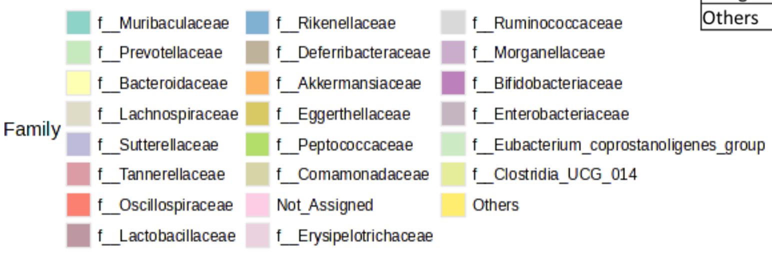

Percent relative abundance (%)

Family	CM (stock)	GMM (stock)
Muribaculaceae	74.28	78.59
Prevotellaceae	13.48	3.48
Lachnospiraceae	2.45	3.76
Sutterellaceae	1.68	1.80
Tannerellaceae	1.64	1.18
Bacteroidaceae	1.49	5.11
Oscillospiraceae	1.00	0.76
Deferribacteraceae	0.63	0.34
Rikenellaceae	0.57	0.56
Lactobacillaceae	0.48	0.95
Erysipelotrichaceae	0.46	0.06
Peptococcaceae	0.33	0.40
Not_Assigned	0.29	0.33
Ruminococcaceae	0.27	0.18
Comamonadaceae	0.25	0.38
Eggerthellaceae	0.25	0.53
Eubacterium_coprostanoligenes_gp	0.15	0.01
Akkermansiaceae	0.07	0.86
Clostridia_UCG_014	0.07	0.02
Enterobacteriaceae	0.07	0.11
Bifidobacteriaceae	0.04	0.15
Morganellaceae	0.02	0.42
Others	0.02	0.02
