# Supplementary material for: Giardia spp.-induced microbiota dysbiosis disrupts intestinal mucin glycosylation
Source: Gut Microbes. 2024 Oct 16;16(1):2412676. doi: 10.1080/19490976.2024.2412676 (PMC11485787; doi:10.1080/19490976.2024.2412676)
Supplement: Supplemental Material [file KGMI_A_2412676_SM4170.zip › Supplementary_Material__17_/Supplementary Tables.docx]

**Supplementary Table 1:** Primer sequences for quantitative PCR on mouse tissues

| Primer | Forward Sequence (5’-3’) | Reverse Sequence (5’-3’) | Reference |
| --- | --- | --- | --- |
| Mouse Muc2 | GAA GCC AGA TCC CGA AAC CA | CCA GCT TGT GGG TGA GGT AG | Amat et al, 2017 |
| Mouse C1GalT1 | TCC CTG CCC TTC GTT GAC | CCT ATT GCT GAT CCA CAG AGG | Solatycka et al, 2012 |
| Mouse C2GnT1 | GCC GAT CCA GCA CTC TTT C | CCA AGT TCC TAT CAA GCA CCA G | Solatycka et al, 2012 |
| Mouse C2GnT2 | ATT GCG TAC TCC ATG GTG GTG | CAT ACT GTT CTG CCC TTT CA | Solatycka et al, 2012 |
| Mouse C2GnT3 | GCC GCT GTT CTT GCT GTT TTG | AGT CAC TTG TCA TCG CCA CGA | Kober et al, 2014 |
| Mouse C3GnT | GGC CAG ATT CTC CTC TCT CAA ACG | AGT GCT CCG CTG TCC AGT CCA | Wang et al, 2016 |
| Mouse Fut2 | AGT CTT CGT GGT TAC AAG CAA C | TGG CTG GTG AGC CCT CAA TA | Meng et al, 2007 |
| Mouse Chst4 | TGG GCC CTG AGG AAG CCT AA | GCC TCC TGG ACT CCT CCC TCT | Meng et al, 2007 |
| Mouse St6Gal1 | GCA GGA TCT CTG AAG AAC TCC C | TTG TGC CCA CAT CCT GTT GGA AG | Origene |
| Mouse St6GalNac1 | CGG GAG ATA GAC AGC CAT GAC T | GTG AAG CCA TAG AAG GAT GTC CG | Origene |
| Mouse St3Gal1 | ACC TGA AGG ACT CCT CGT ATG G | GGA AGC TCT CAG GAT ACA CGA G | Origene |
| Mouse St3Gal4 | TGC CTC CAA CAA GAA GCA GAC C | AGC ATC CGC TTG ATG GCG ATA G | Origene |
| Mouse St3Gal6 | GGT GGT TGG TAA TGG AGG AGT G | GCC TGA AGG TTG TTC TTG TCC C | Origene |
| Mouse Klf4 | CTA TGC AGG CTG TGG CAA AAC C | TTG CGG TAG TGC CTG GTC AGT T | Origene |
| Mouse Beta-actin | GAC CAG CGC AGC GAT ATC | CTG TCG AGT CGC GTC CAC | Solatycka et al, 2012 |

**Supplementary Table 2:** Primer sequences for qPCR on human cells

| Primer | Forward Sequence (5’-3’) | Reverse Sequence  (5’-3’) | Reference |
| --- | --- | --- | --- |
| Human Muc2 | CAG CAC CGA TTG CTG AGT TG | GCT GGT CAT CTC AAT GGC AG | Amat et al, 2017  Iwashita et al, 2003 |
| Human beta-actin | CCA ACC GCG AGA AGA TGA C | GGA AGG AAG GCT GGA AGA GT | Amat et al, 2017 |
| Human beta-microglobulin | TTC TGG CCT GGA GGC TAT C | TCA GGA AAT TTG ACT TTC CAT TC | Amat et al, 2017 |
| Human GAPDH | AAG ATC ATC AGC AAT GCC TCC TGC | ATG GAC TGT GGT CAT GAG TCC TTC | Chik et al, 2014 |
| Human C1GalT1 | AAG CAG GGC TAC ATG AGT GG | GCA TCT CCC CAG TGC TAA GT | Chik et al, 2014 |
| Human Chst5 | CCC AGT GAG GAA CTG GTC TTC | ATC TGT GTT CCA GGA AAG CC | Croix et al, 2011 |
| Human Gal3St2 | TGG GCG GCT TGC AGA GAT A | GCT CTA AGT CCG AGT GCA GGA | Croix et al, 2011 |
| Human Fut2 | ATC ATG ACC ATT GGG ACG TT | GTG CTT GAG TAA GGG GGA CA | Lai et al, 2019 |
| Human St6Gal1 | TGA CGC AGT CCT GAG GTT TA | TTT GTG CCC ACA TCT TGT TG | Graziani et al, 2016 |
| Human St6GalNac1 | CAG AGG CAC AAT CAT GGA AG | GCT GAC TTT TGG GAA TGA GC | Chik et al, 2014 |
